# Artificial Intelligence in Bioengineering: Innovations, Challenges, and Future Directions: A Narrative Review

**DOI:** 10.3390/bioengineering13101204

**Published:** 2026-10-16

**Authors:** Daniele Giansanti

**Affiliations:** Centro IATIS, Istituto Superiore di Sanità, 00161 Roma, Italy; daniele.giansanti@iss.it or daniele.giansanti.dan@hotmail.com

**Keywords:** artificial intelligence, bioengineering, machine learning, biomedical engineering, intelligent systems, digital health, medical imaging, wearable technologies, robotics, digital twins

## Abstract

Artificial intelligence (AI) is rapidly transforming bioengineering, with applications ranging from diagnosis and biomedical imaging to wearable technologies, rehabilitation, biomaterials, biofabrication, and digital twins. This narrative review aims to examine the major innovations, challenges, and future directions of AI-enabled bioengineering, with particular attention to its clinical and translational development. The literature was searched in Scopus and PubMed, with emphasis on systematic reviews and meta-analyses and consideration of other relevant secondary literature across the main bioengineering domains. A total of 57 secondary publications were included in the principal evidence base. The review process was additionally informed by the *ANDJ Narrative Review Checklist* and an established qualification and scoring methodology applied to the selected secondary literature. The reviewed evidence shows a broad expansion of AI applications and increasing integration of intelligent technologies into bioengineering systems. However, important challenges remain, particularly regarding data quality and representativeness, validation and generalizability, reproducibility, safety, human oversight, ethical responsibility, equity, and regulatory requirements. Future developments are expected to increasingly involve multimodal, adaptive, connected, and human-centered systems integrating AI with sensing technologies, robotics, digital twins, and longitudinal data. Overall, AI-enabled bioengineering is progressing from promising technological applications toward increasingly integrated and translational systems. Its successful evolution will depend on combining technological innovation with robust clinical evidence, responsible development, and appropriate ethical and regulatory frameworks.

## 1. Introduction

This review is based on the literature cited throughout the manuscript [1,2,3,4,5,6,7,8,9,10,11,12,13,14,15,16,17,18,19,20,21,22,23,24,25,26,27,28,29,30,31,32,33,34,35,36,37,38,39,40,41,42,43,44,45,46,47,48,49,50,51,52,53,54,55,56,57,58,59,60,61,62,63,64,65,66,67,68,69,70,71,72,73,74,75,76,77,78,79,80,81,82,83,84,85,86,87,88,89,90,91,92,93,94,95,96,97,98,99,100,101,102,103,104,105,106,107,108,109,110,111,112,113,114,115,116,117,118,119,120,121,122,123,124,125,126,127].

Artificial Intelligence (AI) and bioengineering have progressively moved from largely distinct scientific trajectories toward an increasingly integrated research domain. Bioengineering has traditionally applied engineering principles to biological systems, processes, and technologies, supported by computational approaches for modelling, measurement, signal and image analysis, simulation, and system design [1]. Established computational environments and tools used across bioengineering have included statistical software, numerical computing environments such as MATLAB/Simulink (MathWorks, Natick, MA, USA), laboratory and data-acquisition platforms such as LabVIEW (National Instruments/NI, Austin, TX, USA), computer-aided design and engineering (CAD/CAE) systems, and finite-element or multiphysics modelling tools such as ANSYS (Ansys, Canonsburg, PA, USA) and COMSOL Multiphysics (COMSOL AB, Stockholm, Sweden). These approaches provided the basis for quantitative analysis, simulation, optimization, and system development before contemporary AI became widely embedded in bioengineering workflows. The progressive adoption of machine learning and deep learning has subsequently expanded these capabilities by enabling increasingly data-driven approaches to pattern recognition, prediction, classification, optimization, monitoring, and adaptive control [3,4].

A recent development within this trajectory is represented by large language models (LLMs) and related foundation models. Representative examples include ChatGPT/GPT (OpenAI, San Francisco, CA, USA), Gemini (Google, Mountain View, CA, USA), Claude (Anthropic, San Francisco, CA, USA), and Llama (Meta, Menlo Park, CA, USA), together with increasingly specialized biomedical and scientific models. These systems differ in architecture, training strategy, openness, context-handling capacity, and multimodal capabilities, but share the ability to process and generate complex language-based information and, in newer generations, to work with images, audio, code, and external computational tools. In bioengineering and related biomedical contexts, such capabilities may support the extraction and organization of information from scientific literature and technical documentation, synthesis of heterogeneous knowledge, coding and computational assistance, interaction with databases, and processing of multimodal information. Their potential value therefore extends beyond text generation toward supporting the organization, interpretation, and integration of complex datasets and knowledge sources, although their outputs require appropriate domain-specific verification.

Biomechanics represents another important interface between AI and bioengineering. Machine-learning approaches are increasingly used for movement and gait analysis, classification and prediction of biomechanical patterns, estimation of functional and physiological parameters, detection of abnormal motion, and assessment of rehabilitation outcomes. AI can also be combined with wearable sensors, biomechanical measurements, and computational or musculoskeletal models to support patient-specific analysis and adaptive control of prosthetic, orthotic, robotic, and rehabilitation systems [14,15,16]. These applications illustrate how AI can connect data acquisition, biomechanical interpretation, prediction, and technological control within increasingly integrated bioengineering workflows.

The breadth of this convergence is reflected in the rapidly expanding range of bioengineering applications. Machine learning and related AI approaches are being investigated in tissue engineering and regenerative medicine, where they can support the analysis of biological responses, optimization of experimental parameters, and development of engineered tissues and regenerative strategies [5,6]. AI is also being incorporated into three-dimensional bioprinting and biofabrication, including optimization of printing parameters, process monitoring, and development of more reproducible fabrication strategies [7,8]. In parallel, AI-assisted approaches are increasingly being used for the design, prediction, and optimization of biomaterials, creating opportunities to link material properties with biological performance and accelerate materials discovery [9,10].

Other areas of bioengineering are undergoing similar developments. AI-enabled biochips are being investigated for increasingly sophisticated biomedical applications, while the combination of AI with wearable biosensors and smart health devices is expanding the capacity for continuous monitoring and interpretation of physiological and behavioural signals [11,12,13]. In rehabilitation and assistive bioengineering, AI and machine learning are being integrated with robotic systems to support adaptive control and personalized rehabilitation, while related approaches are being explored in prosthetics and orthotics to improve interaction between users and assistive devices [14,15,16]. These developments illustrate a broader shift in which AI is becoming increasingly embedded within physical and biological systems rather than operating exclusively as an external analytical component.

Digital twins represent another important dimension of this evolution. By combining computational models with data derived from biological or clinical systems, digital twins have the potential to support simulation, prediction, monitoring, and individualized decision-making across health and bioengineering applications [17,18]. Their development also illustrates the growing convergence between AI, sensing, modelling, and personalized bioengineering, in which continuously updated computational representations may interact with real-world systems and progressively support more adaptive approaches.

At the frontier of this convergence, AI is increasingly being associated with integrated, autonomous, and closed-loop approaches. The combination of AI-driven engineering biology with emerging technologies is contributing to increasingly interconnected and potentially autonomous biofoundry environments [19]. At the same time, AI-assisted biomimetics is extending computational approaches toward the identification of biological principles and the generation of engineering solutions inspired by biological systems [20]. These developments point toward a transition from isolated AI applications toward more integrated bioengineering ecosystems in which data, algorithms, biological processes, materials, devices, and human users can interact dynamically.

A growing number of recent reviews have examined specific applications of AI across bioengineering and closely related biomedical domains. Recent examples include reviews focused on physical rehabilitation [39], wearable AI-based monitoring [41], head and neck cancer [43], prosthodontics [44], AI-assisted surgery and robotics [45], medical imaging [48], orthopaedics [75], and digital twins in radiology [76]. These contributions provide valuable and increasingly detailed syntheses of individual applications, technologies, or clinical areas. However, their predominantly domain-specific focus makes it more difficult to identify common technological trajectories, cross-cutting challenges, and emerging directions across the broader AI-enabled bioengineering landscape.

The present review addresses this gap through a cross-domain narrative perspective. Rather than focusing on a single application or technology, it brings together representative developments across multiple bioengineering domains and interprets them through three complementary dimensions: **Innovations, Challenges, and Future Directions**. In addition to describing emerging applications, particular attention is given to the maturity of the evidence, validation and generalizability, translational relevance, and the technological, ethical, and regulatory factors that influence progression toward real-world implementation. In this respect, the review also extends the broader conceptual perspective introduced in the *Bioengineering* Editorial “When Bioengineering Met Artificial Intelligence: From Machine Learning to Intelligent Bioengineering Systems” [1], by providing a more structured narrative synthesis of the evolving evidence across domains.

For the purposes of this review, **AI-enabled bioengineering** refers to applications in which AI or machine-learning methods have a substantive role in the analysis, prediction, classification, design, optimization, monitoring, control, personalization, or decision support associated with biological, biomedical, or engineered systems. This definition provides the principal boundary of the review. Technologies are considered within scope when AI represents an active computational component of the bioengineering process, rather than when they merely provide digital or technological support without a meaningful AI component. This distinction is particularly relevant to areas such as digital health monitoring, biomedical and bioengineering imaging, wearable and biosensing systems, biomechanics, rehabilitation, robotics, and intelligent assistive technologies.

The rapid expansion of these applications is accompanied by important challenges. AI-enabled bioengineering systems depend on the quality, representativeness, and interoperability of the data used for their development and validation, while differences between experimental and real-world conditions can affect generalizability and reproducibility [3,4]. Additional concerns include transparency and interpretability, bias, privacy and cybersecurity, standardization, regulatory requirements, and appropriate human oversight. These issues become particularly relevant when AI is integrated into systems that interact directly with biological processes, patients, or assistive technologies, where technical performance must be considered together with safety, accountability, ethical acceptability, and translational value.

Against this background, the Special Issue **“Artificial Intelligence in Bioengineering: Innovations, Challenges, and Future Directions”** provides a timely context for examining the rapidly evolving intersection between AI and bioengineering (see reference [2] for the summary and objectives of the special issue). The diversity of topics encompassed by this field, ranging from biomedical signals and imaging to biomaterials, digital twins, intelligent devices, robotics, rehabilitation, and emerging autonomous systems, further highlights the need for an integrative perspective capable of identifying common developments and cross-cutting challenges across individual application domains.

Accordingly, this article is conceived as a narrative review aimed at providing an integrative and critical perspective on the rapidly evolving relationship between AI and bioengineering, while also providing a broad scientific context for the Special Issue (see reference [2] for the summary and objectives of the special issue). Rather than pursuing an exhaustive or systematic mapping of the literature, the review brings together representative developments from different bioengineering domains to identify major technological advances, cross-cutting challenges, and emerging opportunities. This narrative approach is particularly appropriate for a rapidly evolving and highly heterogeneous field, in which new applications, methodologies, and technological paradigms are continuously emerging.

The review is organized around three complementary dimensions. **Innovations** examines how AI is expanding the capabilities of bioengineering across areas including biomedical signals and imaging, biosensors and wearable technologies, biomaterials and tissue engineering, biofabrication, robotics and rehabilitation, assistive technologies, digital twins, and other emerging intelligent systems. **Challenges** addresses the main factors that may limit the reliability, reproducibility, scalability, and effective translation of AI-enabled bioengineering, including data quality and interoperability, validation and generalizability, explainability and transparency, bias and fairness, privacy and cybersecurity, reproducibility and standardization, and ethical and regulatory requirements. **Future Directions** explores emerging developments that may shape the next generation of AI-enabled bioengineering, including multimodal and increasingly integrated AI approaches, generative and autonomous systems, AI-enabled digital twins, adaptive and closed-loop technologies, human–AI collaboration, personalized bioengineering, and the convergence of computational intelligence with biological and engineered systems.

Through this three-dimensional narrative framework, the review aims to provide a broad, critical, and forward-looking perspective on AI in bioengineering, highlighting **what is changing, what currently limits progress, and what emerging developments may shape the field in the future**.

## 2. Review Design and Organization

### 2.1. Design of the Review

This article was designed as a **narrative review** focusing on the evolving role of artificial intelligence (AI) across major areas of bioengineering. The review was developed to provide an integrated interpretation of current innovations, emerging challenges, and future directions, with particular attention to technological development, biomedical applications, translational aspects, and clinical relevance.

The methodological and reporting process was additionally informed by the *ANDJ Narrative Review Checklist* [21], which provides recommendations for transparent reporting of narrative reviews. The application of the checklist to the present review is described in Supplementary Material S1.2, with the individual checklist items reported in Table S1.

An additional qualification and scoring methodology was applied to the secondary literature considered for the narrative synthesis. This methodology was previously developed and applied in a series of narrative and umbrella reviews [122,123,124,125,126,127]. In the present review, it was adopted as an additional methodological component to appraise selected secondary publications according to predefined methodological and reporting parameters. The parameters, scoring system, and qualification thresholds are detailed in Supplementary Material S1.1.

The literature identification process was organized around three complementary thematic areas: **Innovations, Challenges, and Future Directions**. For each thematic area, a dedicated search strategy was applied using terms related to AI technologies, relevant bioengineering domains, and the specific thematic dimension. The complete search strategies are reported in Table A1.

The searches were primarily oriented toward the identification of **systematic reviews and meta-analyses**, which constituted the principal within the secondary literature. However, other forms of secondary literature retrieved through the thematic searches were also considered, including scoping reviews, narrative reviews, umbrella reviews, and other particularly structured review articles, when they were relevant to the objectives of the review and provided an informative contribution to the narrative synthesis.

The selection of the literature was guided by its **relevance to the review objectives and thematic scope**, together with consideration of the methodological and reporting characteristics and the potential contribution of each publication to the narrative synthesis. The detailed literature identification and selection process, including the search parameters and the successive stages of selection, is reported in Supplementary Material S1.3.

The distinction between **secondary and primary literature** was based primarily on their role within the review. Secondary publications constituted the principal evidence base for the narrative synthesis, whereas relevant primary studies were considered primarily in Discussion to complement and contextualize the secondary evidence, particularly with regard to clinical evaluation, translational aspects, real-world applicability, and patient-relevant outcomes.

### 2.2. Organization of the Review

The review is organized into three main sections corresponding to the three complementary dimensions identified in the Introduction.

*Section 3—Innovations* examines the principal current and emerging applications of AI across bioengineering. It considers how AI is contributing to analysis, prediction, design, optimization, monitoring, control, and personalization across different domains, including biomedical signals and imaging, biosensors and wearable technologies, biomaterials and tissue engineering, biofabrication and three-dimensional bioprinting, robotics and rehabilitation, prosthetics and assistive technologies, digital twins, and other intelligent bioengineering systems.

*Section 4—Challenges* addresses the principal technological, methodological, ethical, regulatory, and translational issues that may limit the reliability, reproducibility, scalability, and implementation of AI-enabled bioengineering. Particular attention is given to data-related limitations, validation and generalizability, explainability and transparency, bias and fairness, privacy and cybersecurity, standardization, safety, and human oversight.

*Section 5—Future Directions* explores emerging developments that may shape the next generation of AI-enabled bioengineering. These include multimodal and integrated AI, generative and autonomous approaches, AI-enabled digital twins, adaptive and closed-loop systems, human–AI collaboration, personalized bioengineering, and the convergence of computational intelligence with biological, physical, and engineered systems.

Section 6—Discussion provides an integrative interpretation of the evidence presented across the three main dimensions of the review, considering their relationships and the broader technological and translational context. It also discusses the limitations of both the available evidence and the present review, considers selected primary clinical and translational studies alongside the secondary literature, and outlines directions for future research in AI-enabled bioengineering. Section 7—Conclusions summarizes the principal insights emerging from the review and highlights the key considerations for the responsible, reliable, and effective development of AI-enabled bioengineering.

Figure 1 provides an overview of the main results of the review, which are reported and organized into three complementary domains: **Innovations (Section 3), Challenges (Section 4), and Future Directions (Section 5)**. The **Innovations** domain describes the current expansion of AI across robotics, rehabilitation and assistive technologies, wearable and sensing systems, physiological signal analysis, diagnostic and clinical decision processes, specialized bioengineering applications, and intelligent and immersive human–technology interfaces. The **Challenges** domain addresses the main factors limiting the translation and sustainable implementation of AI-enabled bioengineering, including evidence quality and validation, clinical integration, data quality and reproducibility, real-world and longitudinal application, as well as AI-specific issues such as bias, domain shift, explainability, uncertainty, model drift, post-deployment monitoring, privacy, cybersecurity, and human oversight. The **Future Directions** domain describes the expected evolution toward clinically integrated AI systems, advanced medical imaging and predictive medicine, multimodal data integration and connected healthcare infrastructures, personalized and adaptive systems, digital twins, continuous monitoring, and increasingly intelligent human–technology interaction. The three domains are further structured into the subsections presented in Section 3, Section 4 and Section 5, respectively, while the main evidence and characteristics identified for each domain are summarized in Tables 1–3. Together, Figure 1 and the three summary tables provide an integrated representation of the current innovations, major challenges, and future development of AI-enabled bioengineering.

## 3. Innovations

Artificial intelligence is progressively expanding the capabilities of bioengineering systems beyond conventional data analysis, enabling new forms of intelligent monitoring, prediction, personalization, optimization, and automation. The following discussion draws on 17 representative studies [22,23,24,25,26,27,28,29,30,31,32,33,34,35,36,37,38] addressing different applications of AI across bioengineering and healthcare-related domains. The studies considered in this section illustrate this evolution across several interconnected areas, including robotics and rehabilitation, wearable and sensing technologies, computational analysis of biological signals, diagnostic imaging, prosthetic and dental applications, interventional technologies, and immersive systems. Taken together, these developments show how AI is increasingly becoming an active component of bioengineering systems and workflows.

### 3.1. AI, Robotics, Rehabilitation, and Assistive Technologies

The integration of AI with **robotic and assistive systems** represents an important area of innovation. AI-assisted robotic surgery is being investigated for applications including surgical assistance, image and video analysis, precision enhancement, and workflow-related functions, bringing computational intelligence increasingly close to the physical execution of clinical procedures [22]. Similar developments are emerging in rehabilitation, where technology-supported approaches are being explored to provide more individualized and data-informed interventions. A systematic review of postpartum rehabilitation identified artificial intelligence and image-processing approaches among the technologies being investigated, illustrating the progressive integration of computational methods into rehabilitation pathways [23].

AI is also contributing to the optimization of **prosthetic and assistive devices**. Automated approaches to transtibial prosthesis alignment illustrate how computational methods can support the adjustment and optimization of physical devices [27]. In parallel, developments in bionic artificial hands combine sensing, signal processing, control, and machine-learning approaches to improve functionality and interaction between the prosthesis and the user [34]. These applications illustrate a broader transition from conventional assistive devices toward systems capable of incorporating increasingly sophisticated computational functions.

From a **biomechanical perspective**, these developments extend AI beyond the analysis of isolated signals toward the interpretation and optimization of human movement and user–device interaction. In prosthetic and assistive applications, AI can support alignment, functional assessment, control, and adaptation by combining data from sensors and user movements with computational models of system behaviour [27,34]. The integration of deep learning with biomechanical and musculoskeletal models represents a further emerging trajectory, in which data-driven approaches may complement model-based representations to support more personalized simulation, prediction, optimization, and control of rehabilitation, orthotic, and prosthetic systems. This progression points toward increasingly **hybrid and patient-specific bioengineering systems**, in which AI and biomechanical modelling can operate as complementary components rather than as separate approaches.

### 3.2. Wearable Technologies, Intelligent Monitoring, and Physiological Signals

Another important area of innovation concerns the combination of AI with **wearable and sensing technologies**. Smart wearable systems have been investigated for diabetic foot prevention, assessment, and management, with sensors capable of monitoring parameters such as plantar pressure, temperature, humidity, and other variables relevant to foot health [24]. The incorporation of AI into these systems creates opportunities for continuous interpretation of multidimensional data and for the development of more responsive monitoring strategies.

Computational intelligence is also increasingly being applied to **physiological and behavioral signals**. In headache medicine, AI approaches have been investigated for diagnosis and classification, prediction of headache attacks, analysis of neuroimaging and neurophysiological information, and prediction of treatment response [25]. Similarly, computational intelligence has been applied to the detection and classification of stuttering, supporting data-driven approaches to the assessment of speech-related conditions [26]. In cardiovascular bioengineering, AI and electrocardiography are being combined for cardiovascular disease diagnosis, demonstrating the potential of machine-learning approaches to extract clinically relevant information from complex physiological signals [29].

These developments illustrate a progressive shift from episodic or manually interpreted measurements toward **continuous, computationally supported monitoring**, in which wearable and biosensing technologies can generate data that are increasingly suitable for automated analysis and individualized assessment.

### 3.3. AI-Supported Diagnosis and Clinical Decision Processes

Medical imaging represents one of the most established areas of AI-enabled innovation. A systematic review and meta-analysis examining AI for the detection of tumor metastases from medical radiology imaging reported pooled sensitivity of 82%, specificity of 84%, and an area under the curve of 0.90 [28]. These findings illustrate the potential of AI-based approaches to provide quantitative support for complex image interpretation and diagnostic tasks.

The application of AI to imaging and clinical workflows has also expanded in other settings. During the COVID-19 pandemic, digital imaging technologies and AI-based applications were investigated for activities including screening, diagnosis, monitoring, and healthcare management [35]. In interventional cardiology, AI has been investigated for image analysis, procedural planning, intraprocedural guidance, prognostic assessment, and decision support [36]. Together, these developments indicate a progressive movement from AI as a tool for isolated image analysis toward its integration into **broader diagnostic and procedural workflows**.

### 3.4. AI in Specialized Bioengineering Applications

The application of AI to specialized domains further illustrates the breadth of current innovation. In prosthodontics, artificial neural networks have been investigated across a range of clinical and technical applications, demonstrating the incorporation of computational intelligence into dental engineering and clinical workflows [33]. This represents an example of how AI can be adapted to highly specialized domains in which anatomical, functional, and technical information must be integrated.

AI-enabled technologies are also being developed in **different healthcare and infrastructural contexts**. A review of innovative technologies addressing neglected tropical diseases in African settings identified artificial intelligence among the emerging technologies being considered alongside other technology-enabled approaches for diagnosis, surveillance, prevention, and disease management [31]. Similarly, the deployment of virtual healthcare and digital technologies during the COVID-19 pandemic in South Africa included applications involving artificial intelligence, chatbots, and robotics, illustrating how computational technologies can become integrated into healthcare delivery under rapidly changing conditions [32].

These examples emphasize that AI innovation in bioengineering is not limited to highly specialized laboratory environments, but is also influenced by the **availability of infrastructure, healthcare needs, and the context in which technologies are deployed**.

### 3.5. Intelligent and Immersive Human–Technology Interfaces

A further direction of innovation concerns systems designed to interact more directly with users. Immersive wearable virtual reality has been investigated in autism for assessment and intervention, with emerging applications incorporating AI-based approaches alongside immersive and wearable technologies [37]. This convergence creates opportunities for increasingly personalized and interactive systems in which sensing, computational analysis, and user interaction can be combined.

Machine-learning approaches for fall detection and prevention provide another example of the integration of computational intelligence with human-centered sensing systems [38]. Such systems aim to identify potentially hazardous events from sensor-derived information and can contribute to safer environments and more responsive assistive technologies.

### 3.6. Comparative Perspective on AI Approaches, Evidence, and Translational Maturity

Although the reviewed applications are diverse, the selected studies show several recurring patterns in the way AI is incorporated into bioengineering systems. Machine learning and deep learning are the predominant approaches, but their role varies substantially according to the type of data and the intended function of the system. In imaging, deep learning is mainly used for detection, classification, segmentation, and quantitative image interpretation [28,35]. In physiological and behavioral applications, machine-learning approaches are more frequently applied to signal classification, prediction, pattern recognition, and continuous monitoring [25,26,29,38]. In robotics, prosthetics, and assistive technologies, AI is more closely connected with control, optimization, alignment, and interaction with physical devices [22,27,34]. This distinction is relevant because the technological complexity and validation requirements of an AI model may differ considerably depending on whether it provides an analytical output, supports a clinical decision, or directly influences the operation of a physical system.

The type of input data also represents an important differentiating factor. Imaging-based applications generally rely on relatively structured and standardized datasets, whereas wearable, physiological, behavioral, and assistive applications often involve continuously acquired, multimodal, and context-dependent data [24,25,26,29,30,37,38]. This creates different challenges for model development and validation. Systems based on continuous or multimodal data may potentially provide more personalized and longitudinal information, but they are also more sensitive to variations in sensors, acquisition conditions, user behavior, and real-world environments. Conversely, high diagnostic performance obtained from controlled imaging datasets does not necessarily demonstrate equivalent performance when systems are transferred to different populations, devices, or clinical settings [28].

The level of evidence also varies considerably across the reviewed domains. Some applications are supported by systematic reviews and meta-analyses reporting quantitative diagnostic performance, such as AI-based detection of tumor metastases [28], whereas other areas are represented mainly by heterogeneous collections of exploratory or early-stage studies [23,26,27,30,34,37]. The presence of quantitative performance measures should therefore not be interpreted as equivalent to clinical validation. Sensitivity, specificity, classification accuracy, or other model-level metrics describe technical performance under the conditions investigated, while clinical translation additionally requires external validation, reproducibility, integration into workflows, assessment of user interaction, and evidence of benefit in real-world settings.

A similar gradient can be observed in translational maturity. AI-supported diagnostic imaging and some cardiovascular applications are relatively close to established clinical workflows, whereas AI integrated with prosthetic control, immersive systems, wearable monitoring, and automated physical-device adjustment remains more dependent on prototype development and contextual validation [22,24,27,34,36,37,38]. The studies therefore suggest that innovation should not be understood only in terms of increasingly sophisticated algorithms. In several bioengineering applications, the more important step is the integration of AI with sensors, physical devices, clinical workflows, and human–technology interaction.

Across the reviewed evidence, three broad levels of AI integration can consequently be identified. At the first level, AI primarily performs data interpretation, such as image or signal classification [25,26,28,29,33,35]. At the second level, AI contributes to prediction, monitoring, optimization, or decision support [24,27,30,36,38]. At the third level, AI becomes integrated with physical or interactive systems, including robotic surgery, prosthetic technologies, and immersive environments [22,34,37]. This progression illustrates a movement from AI as an analytical component toward AI as an active component of bioengineering systems. At the same time, the closer AI becomes to physical intervention or clinical decision-making, the greater the requirements for validation, reliability, safety, interpretability, and human oversight.

### 3.7. Scope and Coverage of AI-Enabled Bioengineering

The selected studies also illustrate an important characteristic of AI-enabled bioengineering: the field is highly heterogeneous, and not all traditional bioengineering domains are equally represented in the available evidence considered here. The present selection provides stronger coverage of robotics and assistive technologies, rehabilitation, wearable and sensing systems, physiological signals, medical imaging, prosthetic and dental applications, interventional technologies, and immersive human–technology interfaces [22,23,24,25,26,27,28,29,30,31,32,33,34,35,36,37,38]. These areas share a relatively direct interaction between AI methods, biological or clinical data, engineered systems, and end users.

Other established bioengineering areas, including tissue engineering, regenerative medicine, biomaterials, bioprinting, and biochips, are not examined with the same depth in the selected evidence base. Rather than extending the review through the addition of further studies solely to obtain a more balanced distribution across traditional bioengineering domains, these areas are considered outside the principal analytical focus of the present section. The review therefore emphasizes the interface between AI, biological information, engineered devices, sensing systems, clinical processes, and human–technology interaction.

This delimitation is important for interpreting the breadth of the review. The objective is not to provide an exhaustive survey of every bioengineering application in which AI may potentially be used, but to illustrate representative pathways through which AI is currently contributing to bioengineering innovation. Within this perspective, the selected studies provide examples of how similar computational approaches can be adapted to substantially different systems, from medical images and physiological signals to robotic devices, prostheses, wearables, and immersive technologies.

Overall, the comparison across the selected studies indicates that the main innovation is not simply the adoption of AI within individual bioengineering domains. Rather, it lies in the increasing coupling of AI with sensing, physical systems, multimodal data, and clinical or user-centered workflows. The resulting systems differ substantially in technological maturity and evidentiary support, and their translational potential depends not only on model performance but also on validation, interoperability, usability, safety, and integration into real-world environments.

Overall, the 17 selected studies [22,23,24,25,26,27,28,29,30,31,32,33,34,35,36,37,38] illustrate a broad but increasingly interconnected landscape of AI-enabled innovation in bioengineering. The main applications, AI approaches, evidence characteristics, limitations, and translational perspectives identified across the selected review studies are summarized in Table 1.

Current applications span robotics and surgery, rehabilitation, wearable monitoring, physiological signal analysis, diagnostic imaging, prosthetic and dental technologies, interventional procedures, context-specific healthcare applications, and immersive human–technology interfaces. Across these domains, the role of AI is progressively extending beyond conventional data analysis toward classification, prediction, optimization, monitoring, personalization, and decision support. Innovation in bioengineering therefore does not follow a single technological pathway, but increasingly reflects the convergence of computational intelligence with biological information, sensing technologies, physical devices, clinical workflows, and human–technology interaction.

The “translational status” categories reported in Table 1 represent an author-derived qualitative assessment rather than standardized classifications directly assigned by the cited studies. The assessment considers the reported level of technological development and validation, the nature and maturity of the available evidence, and the extent to which the application has progressed toward clinical or practical implementation. These categories are intended to provide a descriptive indication of translational maturity and should not be interpreted as formal regulatory, clinical-development, or implementation classifications.

## 4. Challenges

Despite the rapid expansion of artificial intelligence across bioengineering and healthcare, its translation into reliable and routinely deployable systems remains associated with several challenges. The following discussion draws on 20 representative references [39,40,41,42,43,44,45,46,47,48,49,50,51,52,53,54,55,56,57,58] addressing different aspects of AI implementation, validation, generalizability, and clinical translation. The available evidence indicates that these challenges concern not only the technical performance of AI models, but also the quality and representativeness of data, methodological robustness, clinical validation, integration into existing workflows, human factors, and the broader organizational and ethical context in which AI-enabled technologies are implemented.

### 4.1. Evidence Quality, Validation, and Generalizability

One of the first challenges concerns the limited and heterogeneous evidence supporting the clinical implementation of AI-enabled bioengineering technologies. In physical rehabilitation, although AI-supported solutions have been investigated across applications including app-based systems, robotic devices, gaming systems, and wearables, the evidence base remains heterogeneous and important barriers and facilitators to implementation have been identified [39]. Similar considerations emerge from the literature on AI in nursing, where the potential benefits for education, clinical practice, and workload management are accompanied by challenges related to workforce adaptation, ethical considerations, and preservation of humanistic aspects of care [40]. These findings suggest that technological feasibility alone is insufficient to ensure effective adoption in real-world healthcare environments.

Challenges related to validation and generalizability are particularly evident in AI systems based on wearable and multimodal physiological data. The systematic review and meta-analysis of wearable AI for sleep apnea demonstrated the potential of these systems for detection and severity assessment, while also highlighting the complexity associated with evaluating performance across different wearable technologies, datasets, and clinical conditions [41]. More broadly, the integration of AI into clinical medicine remains constrained by issues concerning evidence quality, clinical applicability, and the transition from experimental development to routine clinical use [42].

The oncology literature further illustrates the difficulty of translating promising AI performance into robust clinical decision support. A systematic review of systematic reviews in head and neck cancer identified limitations affecting the application of AI and machine learning, emphasizing the need for stronger evidence and more rigorous evaluation before these approaches can be considered fully established adjunctive tools in clinical management [43]. Similar concerns are apparent in prosthodontics, where AI applications are expanding rapidly but the available evidence remains characterized by substantial variation in algorithms, datasets, clinical tasks, and evaluation approaches [44]. Such heterogeneity makes direct comparison between studies difficult and complicates the identification of consistently reliable solutions.

### 4.2. Clinical Integration, Data, and Reproducibility

Another important challenge is represented by the complexity of integrating AI with physical clinical systems. In robotic and AI-assisted surgery, technological development increasingly involves automation, image analysis, and decision support, while effective integration within complex procedural environments also requires reliable interaction between computational systems, robotic platforms, clinicians, and patients, including appropriate haptic and sensory feedback [45]. In pre-operative colorectal cancer lymph node staging, AI-based approaches have demonstrated diagnostic potential, but their clinical value depends on appropriate validation and integration into existing diagnostic pathways rather than performance metrics alone [46]. Similar considerations apply to AI-supported dental implant planning, where computational approaches may improve planning accuracy but must operate within clinically appropriate workflows and under professional oversight [47].

The quality and structure of the underlying data constitute another major challenge. Deep-learning applications for pulmonary nodule detection and segmentation have frequently relied on publicly available datasets, with substantial variation in data sources and methodological approaches [48]. This dependence on selected datasets raises broader questions regarding representativeness, data heterogeneity, and the extent to which model performance obtained in controlled research environments can be generalized to different populations, institutions, imaging protocols, and clinical settings. Consequently, high reported performance does not necessarily guarantee equivalent performance when systems are transferred to new environments.

Implementation represents an additional and broader challenge. A systematic review specifically addressing barriers to AI implementation in healthcare identified multiple obstacles that extend beyond algorithmic performance, including organizational, technical, professional, and contextual factors [49]. These findings reinforce the view that successful AI implementation requires changes in infrastructure, workflows, competencies, and organizational processes. The comparison between deep-learning systems and healthcare professionals provides a further methodological perspective: although pooled diagnostic performance may be comparable in selected settings, relatively few studies included appropriate external validation or direct comparison with clinicians using the same samples, while inadequate reporting limited the interpretation of reported performance [50].

The challenges become particularly relevant when AI is applied to complex and clinically heterogeneous conditions. In cancer-related malnutrition, AI has been investigated for assessment, prediction, and management, but the diversity of clinical contexts, patient characteristics, outcomes, and methodological approaches complicates the development of broadly transferable models [51]. More generally, the comparison of deep-learning systems with healthcare professionals in medical imaging showed that, despite potentially comparable diagnostic performance, relatively few studies provided externally validated results and poor reporting was common [52]. This highlights the distinction between demonstrating technical performance and establishing robust clinical effectiveness.

A further concern is the methodological quality and reliability of the available evidence. A systematic review and meta-analysis of AI-based diagnostic accuracy in digital pathology found that, although high diagnostic performance was reported, 99% of the included studies had at least one domain with a high or unclear risk of bias or concerns regarding applicability [53]. The review also identified frequent ambiguities or omissions in the reporting of case selection, dataset partitioning for model development and validation, and raw performance data. These findings highlight the distinction between promising diagnostic performance and robust evidence supporting clinical use. They also reinforce the importance of critical appraisal, transparent reporting, and rigorous validation when synthesizing evidence in AI and bioengineering.

Methodological and reproducibility issues are also relevant in emerging computational approaches. Deep-learning-based compressed-sensing methods for accelerated MRI reconstruction have attracted substantial research interest, but the evaluation of these approaches involves multiple technical variables related to reconstruction quality, acquisition conditions, datasets, and model architecture [54]. Similar challenges arise in coronary anatomy imaging, where deep-learning methods are being evaluated across different imaging modalities, anatomical targets, and clinical tasks [55]. Variability in datasets, imaging protocols, architectures, and performance measures can make comparisons between studies difficult and may limit the reproducibility and generalizability of reported results.

### 4.3. Translation to Real-World and Longitudinal Applications

The same issues are evident in AI-enabled prenatal ultrasound. Although systematic evidence supports the potential of AI for detecting fetal cardiac abnormalities, the translation of these approaches into clinical practice requires consideration of differences in image acquisition, operator expertise, patient characteristics, and clinical environments [56]. These factors illustrate how AI systems may be sensitive not only to the underlying pathology but also to the conditions under which data are generated and interpreted.

Finally, the growing integration of AI into continuously monitoring and wearable systems introduces additional challenges related to longitudinal data, reliability, and clinical decision-making. AI-integrated smartwatches have been investigated for the early detection of stroke and hemorrhage, illustrating the potential of continuous monitoring but also the need to establish how algorithmic outputs should be interpreted and incorporated into clinical pathways [57]. Similar considerations apply to deep-learning approaches for electrocardiogram analysis, where the large diversity of ECG signals, datasets, algorithms, and clinical applications creates challenges for consistent evaluation and generalizability across populations and settings [58].

Overall, the evidence from these studies indicates that the principal challenges of AI-enabled bioengineering extend well beyond the development of increasingly sophisticated algorithms. Data quality and representativeness, methodological heterogeneity, external validation, reproducibility, clinical integration, professional acceptance, human oversight, and the reliability of the underlying evidence all influence whether promising AI technologies can achieve sustainable real-world impact. Addressing these challenges will therefore require a shift from demonstrating what AI can technically accomplish toward establishing under which conditions AI-enabled bioengineering systems can operate safely, reliably, transparently, and effectively in real-world contexts.

### 4.4. AI-Specific Challenges: Bias, Domain Shift, Explainability, and Uncertainty

Beyond the general issues of evidence quality and clinical validation, the reviewed literature highlights several challenges that are intrinsic to AI-based systems. A first concern is **dataset bias and representativeness**. AI models learn from the data used during development, and limitations in population composition, data acquisition, labeling, or clinical setting may therefore be reflected in model behavior [42,48,51]. A model trained predominantly on selected populations or datasets may not perform in the same way when applied to populations with different demographic, clinical, technological, or environmental characteristics. This is particularly relevant in bioengineering applications involving wearables, imaging, and physiological signals, where differences between devices, acquisition protocols, and user populations can directly affect the input data [41,48,55,58]. Concrete examples from the reviewed literature illustrate these limitations. In a systematic review of deep-learning applications for pulmonary nodule detection and segmentation, only 2 of 9 studies (22%) conducted external validation, and only 2 of 9 studies (22%) were considered to have a low risk of bias across all four QUADAS-2 domains [48]. Similarly, a systematic review comparing artificial intelligence with healthcare professionals found that only 9 of 81 non-randomized studies (11%) were prospective, while 58 of 81 studies (72%) were classified as having a high overall risk of bias [50]. These findings illustrate how limited external validation, study design, and methodological quality can constrain the assessment of generalizability and clinical reliability.

Closely related to this issue is **domain shift**. The distribution of data encountered after deployment may differ from that observed during model development because of changes in imaging equipment, sensors, clinical workflows, patient populations, or environmental conditions [41,48,55,56,58]. Consequently, external validation should not be considered simply as an additional performance test, but as an important step for determining whether a model retains useful behavior outside the environment in which it was developed. This distinction is particularly important for bioengineering systems intended for use across institutions or with different devices.

**Explainability and uncertainty** represent another important aspect. Many AI systems, particularly complex deep-learning models, can provide accurate predictions without making the reasoning behind those predictions readily understandable to clinicians or users [42,44]. This creates difficulties when an AI output needs to be questioned, interpreted in context, or reconciled with other clinical information. In physiological signal analysis, particularly electrocardiography, practical explainable AI approaches have been investigated to identify the signal features contributing to model predictions, including **Grad-CAM, SHAP (Shapley Additive Explanations), and attention mechanisms** [29]. These approaches may improve transparency and facilitate interpretation of AI outputs, although their usefulness depends on the fidelity, stability, and clinical relevance of the explanations provided. In medical imaging, explainability is similarly relevant to the interpretation and validation of complex models, although the selected literature does not provide a sufficiently homogeneous basis for comparing individual XAI methods across imaging applications [42,48]. At the same time, model predictions may contain uncertainty arising from limited or heterogeneous training data, unusual inputs, or differences between development and deployment environments. For clinical and bioengineering applications, therefore, reporting a prediction without an indication of its reliability may be insufficient for appropriate decision-making. Explainability should consequently be considered together with uncertainty assessment and human oversight rather than as an isolated technical property.

**Reproducibility** is also affected by the rapidly changing computational environment in which AI systems are developed. Differences in datasets, preprocessing procedures, architectures, hyperparameters, evaluation metrics, and software or hardware environments can contribute to differences in reported results [44,48,54,55]. This makes transparent reporting and, where possible, the availability of sufficiently documented datasets, code, model specifications, and evaluation procedures important for independent verification. The problem is particularly relevant when apparently similar AI approaches are compared across studies using different datasets or experimental conditions.

Finally, AI-specific risks extend to **privacy and cybersecurity**. Bioengineering systems increasingly process clinical records, medical images, physiological signals, behavioral information, and continuously acquired wearable data [40,41,49,57,58]. The combination of large-scale data collection and networked devices creates requirements for appropriate data governance, access control, secure data transmission, and protection against unauthorized use or manipulation. These concerns are particularly relevant for connected wearables, remote monitoring, robotic systems, and other technologies that may operate across multiple digital and clinical infrastructures. Thus, privacy and cybersecurity should be considered part of system reliability rather than as separate administrative issues.

### 4.5. Model Drift, Post-Deployment Monitoring, and Sustainable Reliability

A further challenge emerges when AI systems move from development into long-term real-world use. Unlike a conventional static software tool, an AI model may encounter data distributions that change over time. Changes in patient populations, disease patterns, clinical practices, sensors, imaging equipment, or data-generation processes may progressively alter the relationship between input data and the model’s expected output. This phenomenon, commonly described as **model drift**, creates a potential gap between performance measured during development and performance observed after deployment [41,49,55,58].

For this reason, validation should not necessarily be regarded as a one-time event. AI-enabled bioengineering systems intended for sustained clinical or real-world use require mechanisms for **post-deployment monitoring**, including monitoring of performance, data characteristics, error patterns, unexpected outputs, and clinically relevant outcomes. Such monitoring is particularly important for wearable and continuously operating systems, where large volumes of data are generated under changing conditions [41,57,58]. It may also be relevant to imaging and procedural systems when equipment, acquisition protocols, or clinical workflows are modified [45,48,55,56].

Post-deployment monitoring also has a human dimension. Clinicians and other professionals need to recognize situations in which an AI output may be unreliable and determine when additional assessment or human intervention is required [40,42,49]. In this context, human oversight is not simply a safeguard added after model development, but an integral component of the overall system. The long-term reliability of AI-enabled bioengineering therefore depends on the interaction between model performance, data quality, technical infrastructure, professional use, and mechanisms for detecting and managing performance changes.

Overall, the evidence from these studies indicates that the principal challenges of AI-enabled bioengineering extend well beyond the development of increasingly sophisticated algorithms. Data quality and representativeness, dataset bias, domain shift, explainability, uncertainty, model drift, privacy and cybersecurity, methodological heterogeneity, external validation, reproducibility, post-deployment monitoring, clinical integration, professional acceptance, human oversight, and the reliability of the underlying evidence all influence whether promising AI technologies can achieve sustainable real-world impact. Addressing these challenges will therefore require a shift from demonstrating what AI can technically accomplish toward establishing under which conditions AI-enabled bioengineering systems can operate safely, reliably, transparently, and effectively in real-world contexts.

The main challenges related to AI implementation, validation, generalizability, and clinical translation are summarized in Table 2, based on the selected review studies.

## 5. Future Directions

The evolution of artificial intelligence in bioengineering is expected to move progressively beyond the development of isolated predictive models toward more integrated, adaptive, multimodal, and clinically embedded systems. The literature reviewed in this section draws on 20 representative references [59,60,61,62,63,64,65,66,67,68,69,70,71,72,73,74,75,76,77,78] addressing future developments in AI-enabled clinical medicine, medical imaging, predictive modelling, wearable technologies, multimodal data integration, connected healthcare infrastructures, orthopaedics, digital twins, and intelligent human–technology interaction. Taken together, these studies suggest that future progress will depend not only on improvements in algorithmic performance, but also on the integration of AI with sensing technologies, advanced imaging, digital infrastructures, patient-specific computational models, and longitudinal clinical information.

### 5.1. From Predictive Models to Clinically Integrated AI Systems

A first major direction concerns the progressive integration of AI into clinical medicine. Recent evidence on AI for cardiovascular diagnosis illustrates the evolution from conventional machine-learning approaches toward multimodal and increasingly complex systems, including emerging agentic architectures [59]. This broader development suggests that future clinical AI may increasingly combine heterogeneous sources of patient information and support multiple stages of the diagnostic and decision-making pathway rather than focusing on isolated predictive tasks.

In oral squamous cell carcinoma, computational and AI-enabled imaging biomarkers are being investigated for predicting and assessing response to immunotherapy, illustrating the growing integration of imaging-derived information with clinically relevant treatment outcomes [60]. Future research may therefore increasingly focus on models capable of combining imaging, pathological, clinical, and treatment-related information to support more individualized assessment. A related direction is emerging in oral health research, where AI is being investigated in combination with advanced biomaterials and computational approaches for microbiome-associated oral infectious diseases [61]. Future developments may extend this convergence between AI and bioengineering toward more integrated systems in which computational models interact with engineered biological materials and disease-specific environments.

The increasing use of large language models represents another important direction in clinically integrated AI. Systematic evidence on medical reasoning with large language models highlights the development of different reasoning strategies, together with challenges related to reliability, clinical integration, and evaluation [62]. Future research should therefore move beyond demonstrating isolated model capabilities and increasingly assess how such systems can be safely integrated into clinical workflows, decision-support processes, and interactions with healthcare professionals.

### 5.2. AI, Predictive Medicine, and Advanced Medical Imaging

Medical imaging is likely to remain one of the major areas of future AI development. Recent work on AI applications using patient-generated health data in pre-care processes illustrates a broader movement toward combining information generated by patients with computational approaches to support healthcare pathways before formal clinical encounters [63]. Future systems may increasingly integrate patient-generated, clinical, imaging, physiological, and longitudinal information to generate more comprehensive patient-specific profiles and support earlier and more personalized interventions.

The development of AI-assisted MRI applications represents another important direction. A systematic review of MRI-based AI in spinal metastases highlights the growing use of computational approaches for the detection, assessment, and characterization of metastatic spinal disease [65]. Similarly, AI-based medical imaging has been investigated for the diagnosis of osteonecrosis of the femoral head, with systematic evidence evaluating diagnostic accuracy across imaging-based approaches [67]. These developments suggest that future imaging systems may increasingly combine automated image analysis with clinical information and disease-specific characteristics to support earlier diagnosis, quantitative assessment, and individualized clinical decision-making.

Machine learning is also being investigated for prediction in neurological applications. Systematic evidence on machine-learning approaches for stroke mortality prediction illustrates the use of computational models to estimate clinically relevant outcomes and identify prognostic patterns [66]. Future systems may increasingly combine demographic, clinical, imaging, and physiological variables to improve individualized risk stratification and outcome prediction.

Magnetoencephalography represents another promising source of rich physiological information. Evidence from adults with mild traumatic brain injury has identified MEG abnormalities across different studies, supporting continued investigation of MEG-derived biomarkers for characterization and assessment of neurological injury [68]. Future research may increasingly combine advanced neurophysiological acquisition with AI and machine-learning methods to identify clinically meaningful patterns and support individualized assessment, although further validation will be required before routine clinical integration.

Machine learning is also emerging in auditory and neural impairment research [71]. Future applications may extend beyond individual classification tasks toward systems that combine physiological signals, imaging, behavioral information, and patient-reported characteristics. In this context, multimodal AI could provide a more comprehensive representation of complex neurological and sensory conditions.

The use of multimodal medical imaging represents a further important direction. Deep learning has been investigated for addressing missing modalities in multimodal imaging studies [72]. Future systems may therefore become increasingly capable of handling incomplete datasets, reconstructing unavailable information, and integrating heterogeneous imaging sources. Such approaches could improve the robustness of AI-based systems in situations in which complete multimodal examinations are not available.

### 5.3. Data Integration, Connected Healthcare, and Multimodal Bioengineering

The future development of AI-enabled bioengineering will also depend strongly on the ability to integrate heterogeneous and large-scale datasets. Research on big-data analytics in COVID-19 diagnosis illustrates the potential of combining large volumes of health information with computational approaches [73]. Future applications may extend beyond specific disease contexts toward integrated platforms capable of processing clinical, imaging, physiological, and population-level information.

Similarly, the continuing development of healthcare information and communication technologies in pediatrics indicates the expansion of digitally connected healthcare environments [74]. Future AI systems may increasingly operate within these interconnected infrastructures rather than as standalone applications. This transition could facilitate the continuous exchange of information between clinical systems, wearable devices, imaging platforms, patient-facing technologies, and decision-support tools.

The increasing availability of wearable and sensing technologies may further support this evolution. Wearable textile UHF-RFID sensors represent an example of increasingly flexible and unobtrusive sensing technologies capable of collecting physiological and contextual information [64]. The continued integration of such sensing platforms with machine-learning approaches may provide an important technological basis for future intelligent monitoring and bioengineering applications.

AI-enabled wearable sensors have also been investigated for physical ergonomics, demonstrating the convergence of wearable sensing and artificial intelligence for the assessment of human movement and physical demands [77]. Future systems may integrate multiple sensors, contextual information, physiological signals, and behavioral patterns to develop more comprehensive models of individual function and risk. This could support a transition from episodic assessment toward continuous, context-aware, and predictive bioengineering applications.

### 5.4. From Specialized Applications to Personalized and Adaptive Systems

Orthopaedic applications provide another example of the expected transition toward more comprehensive AI-supported systems. The literature on deep learning in orthopaedics indicates expanding applications across different clinical and imaging tasks and identifies several directions for future research [75]. Future developments may increasingly combine imaging, biomechanical information, patient characteristics, and longitudinal outcomes to support more personalized prediction and decision-making.

Stroke-related prediction further illustrates the potential of patient-specific computational modelling. Machine-learning approaches have been investigated for predicting stroke mortality and identifying patterns associated with clinically relevant outcomes [66]. Future systems may increasingly integrate multiple clinical, biological, and imaging variables rather than relying on isolated predictors. Such approaches could contribute to bioengineering systems that better reflect the dynamic interaction between biological function, disease progression, anatomy, and engineered interventions.

Digital twins represent a particularly important future direction because they provide a conceptual framework for integrating heterogeneous information into dynamic representations of individual patients or biological systems. A systematic review of digital twins in radiology identifies an emerging field in which imaging data contribute to patient-specific computational representations [76]. Future developments may extend these models through integration with longitudinal clinical information, physiological monitoring, AI-based prediction, and real-time data streams. This could progressively transform digital twins from predominantly representational models into dynamic systems capable of supporting prediction, simulation, and personalized decision-making.

### 5.5. Continuous Monitoring and Intelligent Human–Technology Interaction

Finally, the increasing combination of wearable sensing and AI is likely to support new forms of continuous monitoring and interaction between individuals and bioengineering systems. Interpretable and explainable AI applied to wearable sensor-based fall-risk assessment illustrates the potential of combining continuous sensing with computational models to identify individuals at increased risk of falls, with specific relevance to prosthetics and orthotics [78]. Future systems may combine multiple sensors with contextual information, physiological signals, and behavioral patterns to improve individualized risk assessment and support responsive assistive technologies.

The combination of these technologies could progressively shift bioengineering from episodic assessment toward continuous and adaptive interaction. Rather than simply generating a single prediction, future systems may continuously update their interpretation of an individual’s condition and adapt their outputs according to changes in context, physiological state, functional status, or clinical risk.

Taken together, the evidence reviewed across these 20 references [59,60,61,62,63,64,65,66,67,68,69,70,71,72,73,74,75,76,77,78] suggests that the future of AI-enabled bioengineering will be characterized by greater integration, personalization, multimodality, continuity, and interaction with the physical and clinical environment. Future systems are likely to increasingly combine AI with advanced imaging, wearable and ambient sensing, connected healthcare infrastructures, predictive modelling, and digital twins. At the same time, methodological advances must be accompanied by stronger prospective and multicenter validation, standardized evaluation, appropriate reporting, and integration into real-world clinical workflows. The future development of AI-enabled bioengineering may therefore depend less on the emergence of isolated new algorithms and more on the creation of interconnected and adaptive systems in which artificial intelligence links heterogeneous data, biological processes, engineered technologies, and human decision-making. The main future directions emerging from the selected review studies are summarized in Table 3.

## 6. Discussion

The discussion is organized around three complementary polarities. The first polarity (Section 6.1) addresses the added value and role of the review, highlighting its integrative narrative approach and cross-domain perspective. The second polarity (Section 6.2) focuses on the interpretation of the underlying primary evidence, considering study designs, levels of validation, and translational maturity. The third polarity (Section 6.3) addresses the limitations of the review and the available evidence, together with future research directions, with particular attention to the need for broader, longer-term, and real-world evaluation of AI-enabled bioengineering systems.

### 6.1. Added Value and Role of the Review

The present review provides an integrative perspective on the rapidly evolving relationship between artificial intelligence and bioengineering. Rather than focusing on a single technology or application, it considers the field across three interconnected dimensions—Innovations, Challenges, and Future Directions—and across multiple bioengineering domains.

Across the reviewed literature, a consistent pattern emerges. AI is increasingly being applied to diverse bioengineering contexts, including medical imaging, robotic and assistive technologies, rehabilitation, wearable and physiological sensing, prosthetics, clinical prediction, and digitally connected healthcare. These innovations demonstrate a progressive expansion from task-specific applications toward systems capable of supporting diagnosis, monitoring, prediction, decision support, and personalization. At the same time, the literature identifies several cross-cutting barriers to translation, including heterogeneous datasets and methodologies, limited external and prospective validation, restricted generalizability, interpretability and reliability concerns, interoperability and data-governance requirements, and challenges related to clinical, organizational, ethical, and regulatory integration. Against this background, the emerging directions identified across the reviewed studies point toward increasingly multimodal and interconnected systems, combining AI with advanced imaging, continuous sensing, patient-generated and longitudinal data, large language models, explainable AI, digital twins, and adaptive human–technology interaction. Taken together, these findings suggest a progressive shift from isolated AI applications toward more integrated, personalized, and context-aware bioengineering systems. A graphical synthesis of the main application domains, cross-cutting limitations, and emerging directions identified across the reviewed literature is provided in Figure 2.

The choice of a narrative review design was intentional and reflects the broad and heterogeneous scope of the topic. Narrative reviews are particularly suited to synthesis and critical interpretation across diverse bodies of literature when the objective is to develop a broader understanding of a complex and rapidly evolving field [79,80,81,82]. In contrast, systematic reviews and meta-analyses are generally designed to address focused questions, while scoping reviews systematically map the extent and characteristics of a defined literature. Here, the aim is to connect evidence across different bioengineering areas and identify common technological trajectories, challenges, and emerging opportunities.

The review also complements the Bioengineering Special Issue, “Artificial Intelligence in Bioengineering: Innovations, Challenges, and Future Directions” (see reference [2] for the summary and objectives of the special issues), by connecting developments that are often considered separately. Its purpose is not to establish a definitive roadmap, but to provide a directional and cross-domain perspective that may support interdisciplinary discussion and identify areas for further investigation.

Overall, the added value of this review lies in its integrative role, complementing more focused systematic, meta-analytic, and scoping reviews while providing a common framework for understanding how AI may progressively become integrated into bioengineering systems and workflows.

### 6.2. Interpretation of the Reviewed Evidence

Although the present review is based on a narrative synthesis of secondary literature, the studies discussed within the selected reviews provide an indication of how AI-enabled bioengineering is being investigated at the primary research level. The references considered in this part of the discussion include randomized controlled trials, prospective comparative and multicenter studies, single-group clinical investigations, and preliminary or technology-oriented studies [83,84,85,86,87,88,89,90,91,92,93,94,95,96,97,98,99,100,101,102,103,104,105,106,107,108,109]. Their characteristics provide an additional perspective on the current maturity of the field and help contextualize the innovations, challenges, and future directions identified in the review.

*Innovations.* At the level of primary research, AI-enabled bioengineering is increasingly being evaluated not only through technical performance measures but also through clinical, functional, and patient-centered outcomes. Randomized controlled trials have investigated AI-supported rehabilitation and telerehabilitation, including video-game-based dysphagia rehabilitation, exercise recognition and prescription, self-management, occupational therapy, and smartphone-supported rehabilitation [84,91,92,93,96,98,103]. These studies are relevant because they move the evaluation of AI beyond algorithm development and toward assessment of whether AI-supported interventions can produce measurable changes in patient outcomes or rehabilitation processes.

Other primary studies provide evidence from prospective and comparative designs. A prospective comparative study evaluated a smartwatch and machine-learning system for home rehabilitation in chronic stroke survivors [83], while a multicenter study investigated real-time AI-supported image reporting during esophagogastroduodenoscopy [85]. These designs provide evidence under more clinically relevant conditions and illustrate the progressive movement of AI from experimental systems toward technologies intended for use within real clinical or home-based workflows.

Additional studies remain at earlier stages of clinical or technological development. Examples include single-group clinical evaluation of machine learning combined with augmented reality for phantom limb pain [108], preliminary investigations of vibrotactile sensory substitution for vestibular rehabilitation [86], wearable-based prediction systems [97,101], and studies involving gait recognition or physiological parameter estimation [106,107]. These contributions are nevertheless relevant to the innovation dimension because they demonstrate the diversity of approaches through which AI is being coupled with sensing, wearable devices, rehabilitation technologies, and human–technology interfaces.

*Challenges.* The primary evidence also illustrates why technological innovation does not automatically translate into established clinical effectiveness. The available studies differ substantially in sample size, design, comparator, population, duration of follow-up, outcome measures, and validation strategy [83,84,85,86,87,88,89,90,91,92,93,94,95,96,97,98,99,100,101,102,103,104,105,106,107,108,109]. Randomized controlled trials can provide evidence of intervention effects, while prospective and multicenter studies can contribute information on performance in more realistic settings; however, even these designs may remain limited in scale or duration. Conversely, preliminary and single-group studies are useful for feasibility and early clinical evaluation but provide more limited information on generalizability and comparative effectiveness.

This distinction is particularly important for AI systems based on continuous or multimodal data. Wearable and sensor-based applications for seizure detection, cardiovascular protection, fall prevention, physiological monitoring, and digital biomarkers have been evaluated in clinical or multisite settings [88,89,90,99,101]. Their translation nevertheless depends on performance across different users, environments, sensors, and patterns of data acquisition. Similarly, AI-supported rehabilitation systems may demonstrate improvements under the conditions of a controlled study without necessarily establishing their effectiveness across different healthcare settings or patient populations. The primary literature therefore supports the potential of these technologies while also showing the need for broader validation, longer follow-up, reproducibility, and evaluation under routine conditions.

*Future Directions.* The characteristics of the primary evidence suggest a progressive need to move from feasibility and controlled evaluation toward larger, longer, and more representative clinical investigations. The emergence of randomized and multicenter studies indicates that some AI-enabled bioengineering applications are already entering this stage [84,85,91,92,93,96,98,103]. At the same time, newer areas such as digital biomarkers, personalized prediction, digital twins, and continuously adaptive systems remain comparatively less mature and require further empirical validation [94,95,101].

Future research should therefore increasingly connect technological development with clinically meaningful outcomes, appropriate comparators, representative populations, and longitudinal assessment. This is particularly important when AI systems are intended to continuously monitor patients, adapt interventions, or influence clinical decisions.

The international and regulatory guidance considered in this review provides a complementary framework for this progression [110,111,112,113,114,115]. The WHO guidance emphasizes responsible development of AI for health, including human autonomy, safety, transparency, accountability, inclusiveness, and protection of individuals and communities [110]. The UNESCO Recommendation similarly highlights human rights, fairness, transparency, responsibility, and human oversight in the development and use of AI [111]. At the European level, the Artificial Intelligence Act establishes a risk-based regulatory framework addressing aspects such as risk management, data governance, transparency, human oversight, accuracy, robustness, and cybersecurity [112]. These principles are complemented by guidance from the U.S. Food and Drug Administration, Health Canada, and the MHRA concerning AI-enabled medical devices and good machine-learning practices, including the management of AI systems throughout their lifecycle [113,114,115]. In particular, the guidance on predetermined change control plans addresses the need to anticipate and appropriately evaluate modifications in machine-learning-enabled medical devices following deployment [115].

Taken together, these documents show that the transition from promising primary studies to deployable AI-enabled bioengineering systems involves requirements extending beyond algorithmic performance. Clinical validation, reproducibility, data quality, human oversight, cybersecurity, monitoring, and appropriate management of system changes become integral components of responsible translation. This regulatory and governance perspective therefore complements the heterogeneous primary evidence discussed above and reinforces the need to evaluate AI-enabled bioengineering not only as a technological innovation, but as a system that must remain reliable, safe, and appropriately governed throughout its intended use.

Taken together, the primary studies underlying the reviewed literature indicate a heterogeneous but progressive evidence landscape. Some applications remain at the feasibility or exploratory stage, whereas others have reached randomized, prospective, or multicenter clinical evaluation. Reading the evidence in this way complements the secondary-literature synthesis presented in Section 3, Section 4 and Section 5: the Innovations describe the technological developments identified in the literature, the Challenges are reflected in the limitations and validation requirements of the underlying primary studies, and the Future Directions emerge from the need to progress toward larger, more robust, longitudinal, and real-world evaluations.

### 6.3. Limitations and Future Directions

The present review has several limitations that should be considered when interpreting its findings. First, the narrative review design was intentionally selected to accommodate the broad and heterogeneous nature of AI applications in bioengineering. Consequently, the review was not designed to provide an exhaustive or quantitatively structured synthesis of the available evidence, and no meta-analysis or formal quantitative comparison of outcomes was performed. The selection and interpretation of the literature may therefore be influenced by the scope and focus of the review, although efforts were made to include representative evidence across the main bioengineering domains considered.

A second limitation concerns the heterogeneity of the available literature. The studies and reviews considered differ substantially in AI methodologies, data sources, populations, validation strategies, and outcome measures. This heterogeneity limits direct comparison between technologies and makes it difficult to establish common benchmarks for performance and clinical effectiveness. Moreover, the rapidly evolving nature of AI means that algorithms, datasets, and implementation approaches may change considerably over relatively short periods.

A further limitation is the uneven availability of evidence across bioengineering domains. Some areas, particularly rehabilitation, wearables, imaging, and clinical decision support, are represented by a broader body of literature, whereas other areas remain less consistently investigated. Much of the available evidence also remains focused on technical development or controlled evaluation rather than long-term performance in routine clinical and real-world environments.

Future research should therefore move toward larger and more representative populations, prospective and multicenter validation, longer follow-up, standardized reporting, and evaluation under real-world conditions. Greater attention should also be given to clinically meaningful outcomes, external validation, uncertainty estimation, reproducibility, and performance across different populations, institutions, sensors, and acquisition conditions.

Several specific technological trajectories are particularly relevant. **Explainable artificial intelligence (XAI)** is expected to become increasingly important in medical imaging and physiological signal analysis. Recent work has examined saliency maps, attention mechanisms, gradient-based approaches, Grad-CAM, SHAP, LIME, and related techniques for making model outputs more interpretable [116,117]. Future studies should move beyond the simple generation of explanations and assess their fidelity, stability, reproducibility, and clinical usefulness, including whether they genuinely support human decision-making.

Another relevant trajectory is the deeper integration of **deep learning with biomechanical and musculoskeletal models**. Neural-network surrogates have already been investigated to approximate computationally demanding musculoskeletal models and predict internal forces, while more recent work has extended this approach to patient-specific joint-force estimation and implant-alignment applications [118,119]. Further development of such hybrid approaches could combine the computational efficiency of deep learning with the physiological and mechanistic structure of biomechanical models, supporting more personalized simulation, prediction, optimization, and control of orthotic, prosthetic, and rehabilitation systems.

Future bioengineering systems are also likely to become increasingly **multimodal, longitudinal, and adaptive**. The combination of imaging, physiological signals, wearable measurements, clinical information, and mechanistic models may support more comprehensive patient-specific computational representations. In this context, AI-enabled digital twins may progressively evolve toward continuously updated models that integrate multimodal data, simulation, prediction, and personalized decision support [120]. At the same time, adaptive and closed-loop systems will require robust monitoring of model performance, data drift, and changes occurring after deployment, together with appropriate human oversight and lifecycle management.

Finally, **generative and increasingly autonomous AI** may contribute to design optimization, knowledge synthesis, experimental planning, and iterative design–build–test–learn workflows, including emerging self-driving laboratory approaches in biomanufacturing [121]. These developments will require strong safeguards for reliability, traceability, reproducibility, cybersecurity, and human supervision. Overall, future progress will depend on linking technological innovation with robust clinical evidence, XAI and meaningful human interpretation, hybrid mechanistic–data-driven modelling, responsible governance, and continuous evaluation throughout the lifecycle of AI-enabled bioengineering systems.

## 7. Conclusions

Artificial intelligence is increasingly transforming bioengineering across diagnosis, imaging, biosignal analysis, wearables, rehabilitation, robotics, prosthetics, biomaterials, biofabrication, and digital twins. The reviewed evidence indicates a progressive shift from isolated applications toward more integrated, personalized, and intelligent systems.

However, the **Challenges** identified show that technological performance alone is not sufficient for successful translation. Data quality, validation, generalizability, reproducibility, safety, human oversight, ethical responsibility, and regulatory requirements remain central to the real-world implementation of AI-enabled bioengineering.

The **Future Directions** point toward increasingly multimodal, adaptive, connected, and human-centered systems. Their development will require stronger clinical evidence and responsible integration into real-world healthcare. Ultimately, the next phase will depend not simply on whether AI can be incorporated into bioengineering, but **when, where, for whom, and under which conditions it can deliver meaningful, safe, trustworthy, equitable, and sustainable value**.

## Figures and Tables

**Figure 1:**
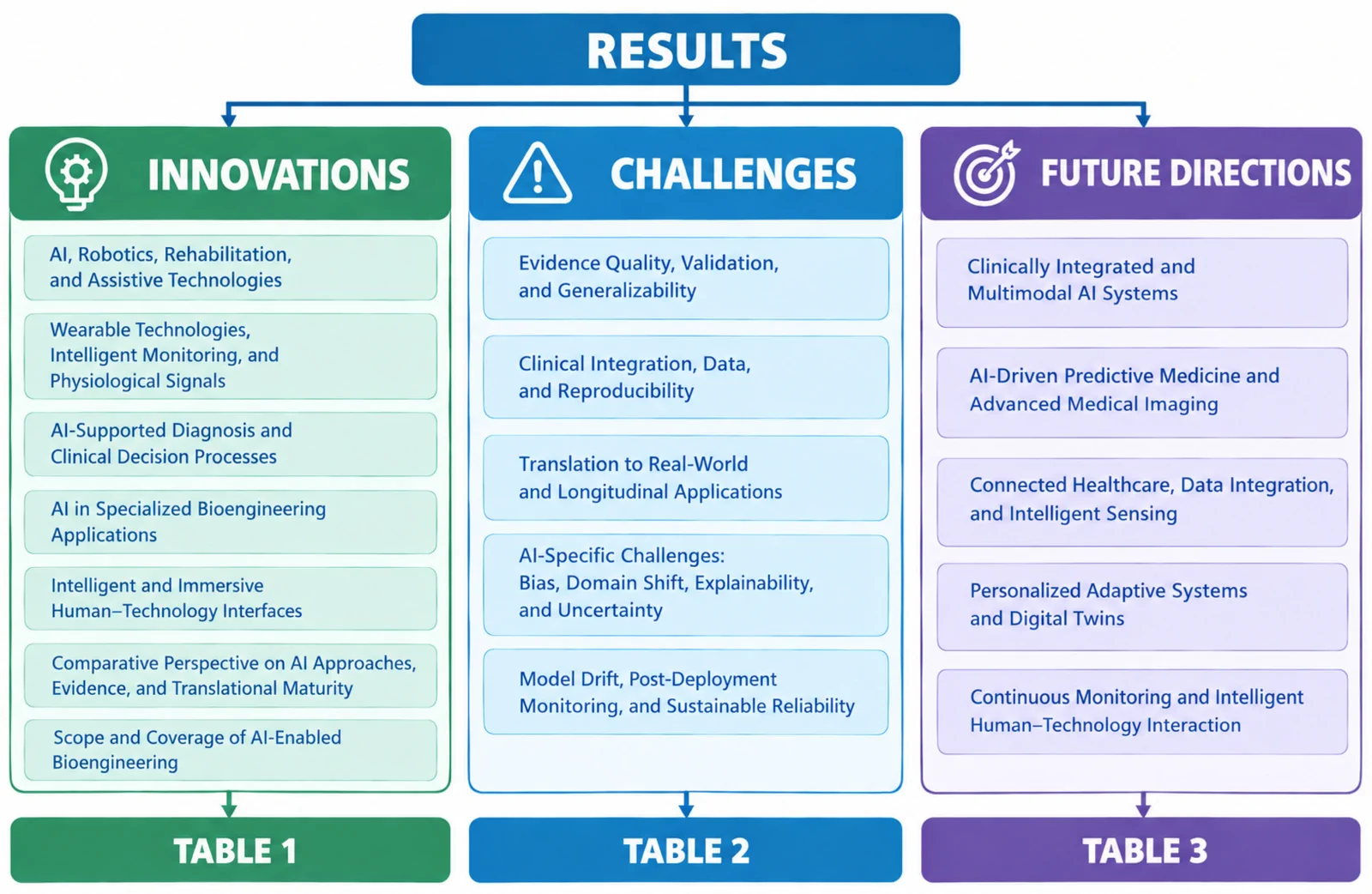
Overview of the main results of the review, organized into innovations, challenges, and future directions of AI-enabled bioengineering. This figure will be provided also with higher resolution.

**Figure 2:**
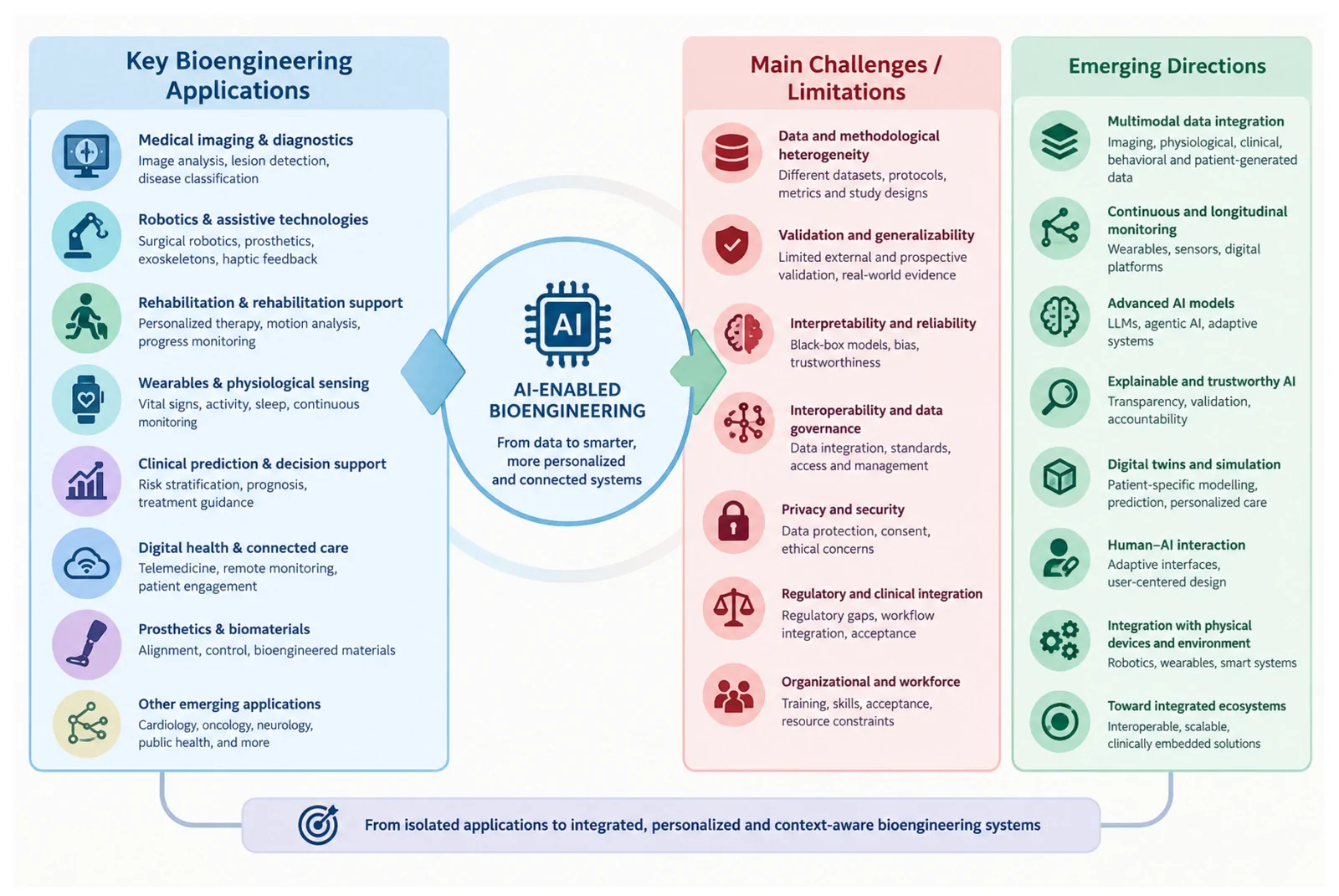
Cross-domain synthesis of AI-enabled bioengineering: major applications, key limitations, and emerging directions. This figure will be provided also with higher resolution.

**Table 1:** Representative evidence on AI-enabled innovations in bioengineering. The table summarizes the main bioengineering applications, AI methodologies and data inputs, key findings and evidence characteristics, major limitations, and the authors’ qualitative assessment of translational status across the representative review studies discussed in Section 3.

Ref.	Bioengineering Application	AI Methodology and Data/Input	Key Findings and Evidence/Validation	Major Limitations	Translational Status
[22]	AI-assisted robotic surgery	AI, machine learning/deep learning; surgical imaging, video, procedural and patient data	AI-assisted robotics is being applied to surgical planning, image/video analysis, precision enhancement, decision support and increasingly automated procedures. The review synthesizes recent clinical and experimental evidence, including comparative outcome data.	High costs, ethical and training issues, need for long-term clinical evaluation and further validation.	Clinical translation/emerging deployment
[23]	Postpartum rehabilitation	AI and image-processing approaches; patient and rehabilitation data, imaging and technology-supported interventions	AI and image-processing approaches are among the technologies investigated in multimodal postpartum rehabilitation. The review reports heterogeneous technology-supported interventions with variable study sizes and durations.	Heterogeneous interventions, limited medical-intervention evidence and variable study designs.	Emerging/research stage
[24]	Diabetic-foot monitoring and management	AI combined with smart wearables and sensing; plantar pressure, shear force, temperature, humidity, vascular/microcirculatory and pH-related parameters	Wearable sensing and AI are presented as promising approaches for continuous monitoring, detection and management of diabetic-foot conditions and wound healing.	Heterogeneity of sensing technologies and need for further clinical validation and real-time implementation evidence.	Emerging; toward continuous clinical monitoring
[25]	AI in headache medicine	Machine learning/deep learning; clinical, neuroimaging, neurophysiological and wearable data	AI has been investigated for diagnosis, headache classification, attack prediction, treatment-response prediction and analysis of neuroimaging/neurophysiological data. The review identifies limited external validation and small or biased datasets in many studies.	Data quality, interpretability, bias, privacy, regulatory gaps and limited generalizability.	Emerging clinical application
[26]	Automated stuttering detection and assessment	Machine learning and computational intelligence; speech/audio signals	AI-based approaches can detect and classify stuttering manifestations and may support assessment and intervention. The review analyzes 14 studies and identifies multiple computational approaches.	Limited evidence base, methodological heterogeneity and need for further development and validation.	Early-stage/emerging
[27]	Automated transtibial prosthesis alignment	Machine learning and automated computational approaches; prosthetic alignment measurements and biomechanical data	The review identifies diverse ML-based and automated approaches for detecting and adjusting transtibial prosthesis alignment, with potential to improve accuracy and reduce human error.	Heterogeneous algorithms and alignment tools; important methodological gaps remain.	Emerging; predominantly research-stage
[28]	AI-assisted detection of tumor metastases	Machine learning and deep learning; medical radiology images	Meta-analysis of 34 studies found pooled sensitivity of 82%, specificity of 84% and AUC of 0.90; ML and DL both showed substantial diagnostic performance.	Variation in study design, imaging modalities, algorithms and datasets; performance does not by itself establish clinical effectiveness.	Advanced research/clinical translation
[29]	AI-enabled ECG analysis	Machine learning, deep learning, CNN, LSTM and hybrid models; ECG signals	AI approaches are applied to detection of heart failure, myocardial infarction and atrial fibrillation, including denoising, synthetic data generation, explainable AI and wearable monitoring.	Dataset and methodological heterogeneity, explainability and implementation challenges.	Emerging to advanced clinical application
[30]	Privacy-preserving aging in place	Machine learning together with sensor-based, wearable, camera-based and robotic technologies; in-home sensor and behavioral data	Smart-home and assistive technologies, including ML approaches, can support independent living while addressing privacy and security requirements.	Privacy, data security, non-intrusiveness and heterogeneous sensing approaches.	Emerging/smart-home research and development
[31]	AI-enabled technologies for neglected tropical diseases	AI alongside other emerging technologies; health, surveillance, diagnostic and population-level data	AI is identified among nine promising technologies for prevention, surveillance, diagnosis, treatment and management of neglected tropical diseases in African settings.	Contextual, infrastructural and sociopolitical constraints; heterogeneous evidence across technologies and applications.	Emerging/context-dependent deployment
[32]	Virtual healthcare and digital health during COVID-19	AI, chatbots and robotics alongside telemedicine, mHealth and other digital technologies; clinical and remote-care data	The review identified AI, chatbots and robotics among technologies deployed during the pandemic in South Africa, alongside telehealth and mobile solutions.	Rapid deployment, heterogeneous technologies and limited evidence concerning longer-term effectiveness and sustainability.	Deployed in practice; variable maturity
[33]	AI in prosthodontics	Artificial neural networks and deep learning; dental/prosthodontic clinical and imaging data	The review mapped 70 reports and identified ANN applications in implant and non-implant prosthodontics, with different data types, architectures and evaluation metrics.	Important limitations across the data lifecycle and substantial heterogeneity in models and evaluation approaches.	Emerging clinical integration
[34]	Bionic artificial hands	Machine learning for gesture recognition; EMG/bioelectrical signals plus tactile, visual and auditory sensory information	ML can improve gesture-recognition accuracy for prosthetic control. The review also identifies advances in sensory feedback and control-system integration.	Integration of control, sensing and mechanical systems remains challenging; further development is required for natural and robust interaction.	Emerging; advanced prototype/research stage
[35]	AI-enabled medical imaging during COVID-19	AI and image-analysis methods; radiological/medical images and clinical information	AI and digital imaging technologies were investigated for screening, diagnosis, monitoring and management during the pandemic.	Heterogeneous applications and rapidly evolving evidence; differences in imaging protocols and AI approaches.	Clinical research/selected practical deployment
[36]	AI in interventional cardiology	Machine learning and deep learning; cardiovascular imaging, procedural and clinical data	ML supports arrhythmia detection and plaque characterization, while DL is applied to image interpretation, robotic navigation and catheter tracking; prediction and personalized treatment are also investigated.	Data privacy, transparency, generalizability, regulatory barriers and need for robust data sharing.	Emerging to advanced clinical translation
[37]	Immersive wearable VR and AI in autism	AI-based systems combined with wearable/immersive VR and eye-tracking; gaze, social-interaction and physiological data	Wearable VR can support assessment and intervention; AI-based systems and eye-tracking enable more objective assessment of gaze, interaction and physiological responses.	Small samples, methodological heterogeneity, limited randomized designs and scarce long-term follow-up.	Emerging clinical/rehabilitation application
[38]	Fall detection and prevention	Machine learning; wearable, environmental and motion-sensor data	ML is increasingly used for fall detection and prevention, with reviews comparing datasets, age groups, sensors, algorithms and settings.	High false-alarm rates in conventional approaches, dataset differences and limited generalizability across environments.	Emerging; some applications approaching practical deployment

**Table 2:** Representative review evidence on challenges in AI-enabled bioengineering. Summary of the main applications, AI approaches, evidence and validation issues, limitations, and translational status identified across the selected review studies.

Ref.	Bioengineering Application	AI Methodology and Data/Input	Key Findings and Evidence/Validation	Major Limitations	Translational Status
[39]	AI in physical rehabilitation	ML/DL; rehabilitation, clinical and patient-related data	AI applications span assessment, prediction, monitoring and personalized rehabilitation, but evidence remains heterogeneous across applications and study designs.	Limited standardization, heterogeneous methods and need for stronger clinical validation and generalizability.	Emerging/research-to-clinical translation
[40]	AI in nursing and healthcare practice	AI/ML; clinical, educational, workload and patient-related data	AI may support clinical practice, education and workload management, but implementation is influenced by professional perceptions and organizational factors.	Skills and training, acceptance, ethical issues, workload implications and implementation barriers.	Emerging clinical integration
[41]	AI-enabled wearable detection of sleep apnea	AI/ML; wearable physiological signals	Wearable AI can detect sleep-apnea-related patterns from physiological signals, supporting potentially continuous and non-invasive monitoring.	Variability in devices, algorithms and datasets; need for external and real-world validation.	Emerging/wearable clinical application
[42]	AI integration into clinical medicine	AI/ML/DL; clinical, imaging, physiological and electronic health data	AI offers opportunities for diagnosis, prediction, personalization and clinical decision support, while translation depends on data quality, validation, explainability and integration.	Data quality, bias, interpretability, ethical/regulatory issues and clinical integration.	Emerging to clinical translation
[43]	AI in head and neck cancer	ML/DL; imaging, clinical and pathological data	AI applications include diagnosis, classification, prognosis and treatment-related prediction across head and neck cancer.	Heterogeneity of studies, limited external validation, dataset limitations and challenges in clinical implementation.	Emerging clinical translation
[44]	AI in prosthodontics	AI/ML/ANN; dental, imaging and clinical data	AI is being investigated for diagnosis, treatment planning, prediction and prosthodontic decision support.	Limited datasets, methodological heterogeneity, interpretability and need for clinical validation.	Emerging/research-to-clinical translation
[45]	Haptic feedback in medical robotics	Haptic technologies; robotic systems; sensor-based and multimodal feedback	Haptic feedback can enhance robotic interaction by providing tactile and force information, with potential benefits for surgical precision, manipulation, training and human–robot interaction.	Heterogeneity of technologies and evaluation methods; technical complexity, integration and cost; limited standardization and clinical validation across applications.	Emerging clinical translation
[46]	AI for pre-operative lymph-node staging in colorectal cancer	ML/DL; medical imaging and clinical data	AI approaches show potential for pre-operative lymph-node staging, with quantitative diagnostic performance reported across studies.	Heterogeneity in datasets, imaging protocols and validation; need for external and prospective evaluation.	Clinical research/emerging translation
[47]	AI in dental implant planning	AI/ML; dental imaging and anatomical data	AI can assist implant planning through anatomical analysis, positioning and treatment-planning support.	Variability in datasets and algorithms, limited standardized validation and uncertainty regarding routine clinical integration.	Emerging clinical application
[48]	Deep learning for pulmonary nodule detection and segmentation	Deep learning/CNN; CT and pulmonary imaging data	DL demonstrates potential for automated pulmonary nodule detection and segmentation, but reported performance varies across datasets and approaches.	Dataset variability, methodological heterogeneity, generalizability and need for external validation.	Advanced research/clinical translation
[49]	Implementation of AI in healthcare	AI/ML; clinical and organizational data	The review identifies multiple barriers to AI implementation, extending beyond algorithmic performance to organizational, technical, ethical and regulatory factors.	Data governance, infrastructure, skills, trust, interoperability, regulation and organizational readiness.	Implementation stage/variable across settings
[50]	AI versus clinicians in medical diagnosis	Deep learning; medical imaging and clinical data	Comparison with healthcare professionals highlights the importance of study design, reporting quality, external validation and appropriate interpretation of performance claims.	Poor reporting, methodological weaknesses, limited external validation and risk of overstating model performance.	Research validation/clinical translation
[51]	AI in cancer-related malnutrition management	AI/ML; nutritional, clinical and cancer-related patient data	AI is being explored for screening, prediction, assessment and management of malnutrition in cancer patients.	Limited evidence, heterogeneous methodologies and need for larger and externally validated studies.	Emerging/research stage
[52]	Deep learning versus healthcare professionals in medical imaging	Deep learning; medical imaging data	Meta-analysis found potentially comparable diagnostic performance between DL systems and healthcare professionals in selected imaging tasks; relatively few studies provided externally validated results.	Limited external validation and poor reporting were common across the evidence base.	Research validation/selective clinical translation
[53]	AI in digital pathology	AI-based image analysis; digital pathology images and diagnostic data	A systematic review and meta-analysis evaluated the diagnostic accuracy of AI in digital pathology. Approximately 99% of the included studies had at least one domain with a high or unclear risk of bias or concerns regarding applicability.	Concerns about risk of bias, applicability, and the reporting and validation of diagnostic performance may limit confidence in the reported results.	Research evidence/clinical validation
[54]	Deep-learning-based fast MRI reconstruction	Deep learning; MRI k-space/image data	DL combined with compressed sensing can accelerate MRI reconstruction while aiming to preserve image quality.	Variability in reconstruction methods, datasets and evaluation metrics; need for broader clinical validation.	Advanced research/emerging clinical translation
[55]	Deep learning in coronary anatomy imaging	Deep learning; coronary CT/imaging data	DL approaches have been investigated for automated coronary anatomy analysis and image interpretation, with quantitative performance evaluated across studies.	Heterogeneous datasets and algorithms, limited external validation and challenges in generalizability.	Advanced research/clinical translation
[56]	AI-enabled prenatal ultrasound	AI/ML; fetal ultrasound images and clinical data	AI can support detection of fetal cardiac abnormalities from prenatal ultrasound, with systematic evidence and meta-analytic diagnostic performance available.	Variation in acquisition protocols, datasets and algorithms; need for external and prospective clinical validation.	Emerging clinical translation
[57]	AI-integrated smartwatch monitoring for stroke and hemorrhage	AI/ML; smartwatch-derived physiological and motion-related data	Smartwatch-based AI is being investigated for early detection of stroke- and hemorrhage-related changes, supporting continuous monitoring concepts.	Limited clinical evidence, device and data heterogeneity, false-positive/false-negative risks and need for prospective validation.	Early-stage/emerging
[58]	Deep learning in ECG analysis	Deep learning; ECG signals	DL approaches have been extensively investigated for ECG analysis, including classification and detection of cardiovascular abnormalities.	Heterogeneous datasets, algorithms and evaluation strategies; generalizability and clinical implementation remain challenging.	Advanced research/emerging clinical translation

**Table 3:** Representative review evidence on future directions in AI-enabled bioengineering. Summary of the main applications, AI approaches, future directions, limitations, and translational perspectives identified across the selected review studies.

Ref.	Bioengineering Application	AI Methodology and Data/Input	Key Findings and Future Directions	Major Limitations	Translational Status
[59]	AI for cardiovascular diagnosis	ML/DL; clinical, imaging and multimodal patient data	AI approaches are evolving from conventional ML toward multimodal and increasingly complex systems, including emerging agentic architectures, with potential for more integrated diagnostic support.	Heterogeneity of approaches and datasets, interpretability, validation, and challenges related to clinical integration and reliability.	Emerging clinical translation
[60]	AI-enabled imaging biomarkers in oral squamous cell carcinoma	AI/ML; medical imaging, pathological and clinical data	AI-enabled imaging biomarkers show potential for predicting and assessing immunotherapy response, supporting more individualized treatment assessment and multimodal clinical modelling.	Heterogeneous evidence, methodological variability and need for further clinical and external validation.	Emerging clinical translation
[61]	AI-driven biomaterials for oral infectious diseases	AI/ML; biomaterials, microbiome-related and oral disease data	AI is being explored in combination with biomaterials for microbiome-associated oral infectious diseases, suggesting future convergence between computational intelligence and engineered biological materials.	Complexity of biological systems, methodological heterogeneity and need for stronger experimental and translational validation.	Early-stage/research
[62]	Reasoning with large language models in medicine	Large language models; medical text, clinical knowledge and multimodal information	Future development focuses on improving medical reasoning capabilities and integrating LLMs into clinically relevant tasks and workflows.	Reliability, hallucination, evaluation standards, clinical safety and integration into healthcare workflows.	Emerging/early clinical translation
[63]	AI using patient-generated health data in elective healthcare	AI/ML; patient-generated health data and pre-care information	AI applications using patient-generated health data may support pre-care processes, risk assessment, information integration and more personalized preparation for elective healthcare.	Data quality, heterogeneity, interoperability, privacy and need for validation in real-world healthcare pathways.	Emerging/research-to-clinical translation
[64]	Intelligent wearable sensing	Textile UHF-RFID sensors; wearable and contextual sensing data	Textile UHF-RFID technologies provide a basis for flexible and unobtrusive sensing, with future potential for integration with intelligent data-processing and monitoring systems.	Sensor performance, communication range, power, integration, data management and need for application-specific validation.	Emerging/research and development
[65]	AI for MRI-based assessment of spinal metastases	AI/ML; MRI and clinical imaging data	AI-based MRI approaches show potential for detection and assessment of spinal metastases, supporting future automated and quantitative imaging workflows.	Dataset and methodological heterogeneity, differences in imaging protocols and need for external and prospective validation.	Emerging clinical translation
[66]	Stroke mortality prediction	Machine learning; clinical and outcome data	ML-based models may support prognostic assessment and individualized risk stratification in patients with stroke.	Heterogeneous datasets and models, variable predictive performance and limited generalizability across populations and settings.	Emerging/research-to-clinical translation
[67]	AI-based imaging diagnosis of femoral-head osteonecrosis	ML/DL; medical imaging data	Medical imaging-based AI shows potential for improving the diagnosis of osteonecrosis of the femoral head, supporting automated and quantitative diagnostic assessment.	Variation in imaging modalities, datasets and algorithms; need for external validation and prospective clinical evaluation.	Emerging clinical translation
[68]	MEG-based neurophysiological assessment	Magnetoencephalography; MEG signals and neurophysiological data	MEG abnormalities identified in mild traumatic brain injury provide a potential basis for future computational and AI-assisted characterization of neurological injury.	Limited and heterogeneous evidence, variability in acquisition and interpretation, and need for stronger validation of AI-based applications.	Early-stage/research
[69]	AI-assisted prediction of hemorrhagic transformation after stroke	AI/ML; neuroimaging and clinical data	AI/ML approaches may support prediction of hemorrhagic transformation and improved prognostic stratification after stroke.	Dataset heterogeneity, variable model performance and need for external and prospective validation.	Emerging clinical translation
[70]	MRI-based AI for Alzheimer’s disease detection	ML; MRI brain imaging data	MRI-based ML approaches show potential for automated detection and diagnostic support in Alzheimer’s disease.	Differences in datasets, imaging protocols and algorithms; generalizability and external validation remain important issues.	Advanced research/emerging clinical translation
[71]	Machine learning in auditory neural impairment	ML; auditory, neurophysiological and clinical data	ML may support automated characterization and classification of auditory neural impairments and provide a basis for future computational diagnostic approaches.	Limited evidence, heterogeneous methodologies and need for larger, validated datasets.	Early-stage/emerging
[72]	Multimodal medical imaging with missing modalities	Deep learning; multimodal medical imaging and incomplete datasets	DL approaches may infer or reconstruct missing imaging modalities, potentially improving multimodal analysis when complete examinations are unavailable.	Dependence on available modalities, reconstruction errors, uncertainty and need for robust clinical validation.	Emerging/research stage
[73]	Big-data analytics for COVID-19 diagnosis	Big-data analytics, ML/DL; clinical, epidemiological and diagnostic datasets	Large-scale computational approaches may support the integration and analysis of heterogeneous information for data-driven diagnosis and public-health applications.	Data heterogeneity, interoperability, quality, privacy and challenges in real-world implementation.	Emerging/research-to-practice
[74]	Connected pediatric healthcare ICT	Healthcare ICT and digital technologies; pediatric clinical and health data	Increasingly connected healthcare technologies provide opportunities for integrating digital information and AI into pediatric clinical workflows and services.	Technological heterogeneity, privacy and security issues, interoperability and limited long-term implementation evidence.	Emerging
[75]	Deep learning in orthopaedics	Deep learning; medical imaging, clinical and musculoskeletal data	DL applications are expanding across orthopaedic imaging, diagnosis and prediction, with future opportunities for more automated and personalized decision support.	Dataset limitations, generalizability, interpretability and need for prospective clinical validation.	Emerging to advanced research
[76]	Digital twins in radiology	Digital twins; patient-specific imaging and clinical data with computational modelling	Digital twins provide a framework for patient-specific modelling and may support future simulation, prediction and personalized radiological decision-making.	Technical complexity, data integration, interoperability, validation and unresolved implementation challenges.	Early-stage/emerging
[77]	AI-enabled physical ergonomics	AI/ML; wearable sensor, physiological and movement data	Combining wearable sensing with AI may enable continuous assessment of physical ergonomics, automated risk identification and more personalized interventions.	Sensor variability, data quality, algorithmic heterogeneity and need for real-world validation.	Emerging/practical application potential
[78]	Explainable AI for wearable fall-risk assessment	XAI/ML; wearable sensor and fall-risk data	Interpretable AI applied to wearable sensor data may support individualized fall-risk assessment, with potential relevance to prosthetics, orthotics and assistive technologies.	Sensor and dataset heterogeneity, explainability limitations, generalizability and need for clinical validation.	Emerging/research-to-clinical translation

## Data Availability

No new data were created or analyzed in this study. Data sharing is not applicable to this article.

## Supplementary Materials

The following supporting information can be downloaded at: https://www.mdpi.com/article/10.3390/bioengineering13101204/s1, S1. Additional Methodological Components.

## Appendix A

**Table A1:** Search strategies used for the three main dimensions of the review.

No.	Search Key
1	Innovations: (“artificial intelligence”[Title/Abstract] OR “machine learning”[Title/Abstract] OR “deep learning”[Title/Abstract] OR “generative artificial intelligence”[Title/Abstract] OR “generative AI”[Title/Abstract] OR “large language model*”[Title/Abstract] OR “foundation model*”[Title/Abstract] OR “neural network*”[Title/Abstract]) AND (bioengineering[Title/Abstract] OR “biomedical engineering”[Title/Abstract] OR biofabrication[Title/Abstract] OR “tissue engineering”[Title/Abstract] OR biomaterial*[Title/Abstract] OR “3D bioprint*”[Title/Abstract] OR biosensor*[Title/Abstract] OR wearable*[Title/Abstract] OR “biomedical signal*”[Title/Abstract] OR “medical imaging”[Title/Abstract] OR robotics[Title/Abstract] OR rehabilitation[Title/Abstract] OR prosthe*[Title/Abstract] OR “assistive technolog*”[Title/Abstract] OR “digital twin*”[Title/Abstract] OR “smart health”[Title/Abstract] OR “intelligent system*”[Title/Abstract]) AND (innovation*[Title/Abstract] OR innovativ*[Title/Abstract] OR “technological innovation*”[Title/Abstract] OR “emerging technolog*”[Title/Abstract] OR “emerging innovation*”[Title/Abstract] OR “novel technolog*”[Title/Abstract] OR “novel approach*”[Title/Abstract] OR “next-generation technolog*”[Title/Abstract] OR “breakthrough technolog*”[Title/Abstract])
2	Challenges: (“artificial intelligence”[Title/Abstract] OR “machine learning”[Title/Abstract] OR “deep learning”[Title/Abstract] OR “generative artificial intelligence”[Title/Abstract] OR “generative AI”[Title/Abstract] OR “large language model*”[Title/Abstract] OR “foundation model*”[Title/Abstract] OR “neural network*”[Title/Abstract]) AND (bioengineering[Title/Abstract] OR “biomedical engineering”[Title/Abstract] OR biofabrication[Title/Abstract] OR “tissue engineering”[Title/Abstract] OR biomaterial*[Title/Abstract] OR “3D bioprint*”[Title/Abstract] OR biosensor*[Title/Abstract] OR wearable*[Title/Abstract] OR “biomedical signal*”[Title/Abstract] OR “medical imaging”[Title/Abstract] OR robotics[Title/Abstract] OR rehabilitation[Title/Abstract] OR prosthe*[Title/Abstract] OR “assistive technolog*”[Title/Abstract] OR “digital twin*”[Title/Abstract] OR “smart health”[Title/Abstract] OR “intelligent system*”[Title/Abstract]) AND (challenge*[Title/Abstract] OR limitation*[Title/Abstract] OR barrier*[Title/Abstract] OR risk*[Title/Abstract])
3	Future Directions: (“artificial intelligence”[Title/Abstract] OR “machine learning”[Title/Abstract] OR “deep learning”[Title/Abstract] OR “generative artificial intelligence”[Title/Abstract] OR “generative AI”[Title/Abstract] OR “large language model*”[Title/Abstract] OR “foundation model*”[Title/Abstract] OR “neural network*”[Title/Abstract]) AND (bioengineering[Title/Abstract] OR “biomedical engineering”[Title/Abstract] OR biofabrication[Title/Abstract] OR “tissue engineering”[Title/Abstract] OR biomaterial*[Title/Abstract] OR “3D bioprint*”[Title/Abstract] OR biosensor*[Title/Abstract] OR wearable*[Title/Abstract] OR “biomedical signal*”[Title/Abstract] OR “medical imaging”[Title/Abstract] OR robotics[Title/Abstract] OR rehabilitation[Title/Abstract] OR prosthe*[Title/Abstract] OR “assistive technolog*”[Title/Abstract] OR “digital twin*”[Title/Abstract] OR “smart health”[Title/Abstract] OR “intelligent system*”[Title/Abstract]) AND (“future direction*”[Title/Abstract] OR “future perspective*”[Title/Abstract] OR prospect*[Title/Abstract] OR “emerging trend*”[Title/Abstract])
