# Supplementary material for: Artificial Intelligence in Bioengineering: Innovations, Challenges, and Future Directions: A Narrative Review
Source: Bioengineering (Basel). 2026 Oct 16;13(10):1204. doi: 10.3390/bioengineering13101204 (PMC13701786; doi:10.3390/bioengineering13101204)
Supplement: Supplementary file 1 [file bioengineering-13-01204-s001.zip › bioengineering-4602772-supplementary.pdf]

bioengineering

MDPI

Review

Artificial Intelligence in Bioengineering: Innovations, Challenges, and Future Directions: A Narrative Review

Daniele Giansanti

S1. Additional Methodological Components

S1.1. Qualification Methodology

An additional qualification methodology was applied to the secondary literature considered for the narrative synthesis. This methodology was previously developed and applied in a series of narrative and umbrella reviews [122–127] and has been consolidated through its use across different healthcare and biomedical topics. In the present review, the same approach was adopted as an additional methodological component to provide a consistent basis for the appraisal of the secondary literature selected for the narrative synthesis.

The purpose of this qualification process was to consider not only the thematic relevance of a publication, but also some basic methodological and reporting characteristics that may contribute to its scientific value and interpretability. The approach was particularly useful for distinguishing publications that could provide a sufficiently robust and informative basis for the narrative synthesis from those offering a more limited methodological contribution. The process was applied while maintaining the flexible and interpretative nature of the narrative review.

For each secondary publication, six parameters were assessed:

- **N1:** Is the rationale for the study clearly described in the Introduction?
- **N2:** Is the study design appropriate for the stated objectives?
- **N3:** Are the methods described clearly?
- **N4:** Are the results presented clearly?
- **N5:** Are the conclusions supported and justified by the results?
- **N6:** Did the authors disclose all relevant conflicts of interest?

Parameters N1–N5 were scored on a five-point scale, from **1 (minimum)** to **5 (maximum)**. Parameter N6 was assessed using a binary **Yes/No** criterion.

For inclusion in the qualified secondary literature, **N6 was required to be "Yes"**, while each of the parameters **N1–N5 was required to receive a score greater than 3**. Publications meeting these criteria were considered suitable for inclusion in the narrative synthesis.

This qualification methodology was therefore used as an additional layer of methodological appraisal alongside the thematic and scientific relevance of the literature. It was not intended to provide a formal risk-of-bias assessment or to establish the exhaustive eligibility of studies, but rather to support a transparent and consistent selection of informative secondary literature for the purposes of the narrative review.

Academic Editor: Hwa Liang Leo

Received: 17 September 2026
Revised: 7 October 2026
Accepted: 8 October 2026
Published: 16 October 2026

Copyright: © 2026 by the author. Licensee MDPI, Basel, Switzerland. This article is an open access article distributed under the terms and conditions of the Creative Commons Attribution (CC BY) license.

S1.2. ANDJ Narrative Review Checklist

As an additional methodological component, the review process was assessed using the *ANDJ Narrative Review Checklist* [21]. The checklist was developed specifically to support the transparent reporting of narrative reviews and covers the main components of the review, from the identification of the literature to the presentation and interpretation of the evidence.

The checklist includes recommendations concerning the identification of the review as a narrative review, the definition of the background and objectives, the description of the literature search and selection process, and the narrative presentation of the reviewed evidence. It also considers the discussion of limitations and quality of the literature, as well as the implications for clinical practice, policy, and future research.

In the present review, the checklist was used as an additional reference to verify that the main methodological and reporting elements expected for a narrative review were appropriately addressed. In particular, attention was given to the transparency of the literature identification process, the definition of the review objectives, and the description of how the selected literature was considered and synthesized.

The individual items of the *ANDJ Narrative Review Checklist*, together with their application to the present review, are summarized in **Table S1**, including the corresponding information on how each checklist item was addressed in the manuscript and/or Supplementary Materials.

Table s1 ANDJ Narrative Review Checklist

**Section / topic**	#	**Checklist item**	**Requirement**	**Reported on**
TITLE	1	Title	Identify the report as a Narrative Review of …	*Title.* The title explicitly identifies the article as a Narrative Review and specifies its focus on AI in bioengineering.
ABSTRACT	2	Unstructured summary	Provide an unstructured summary including, as applicable: background, objective, brief summary of narrative review and implications for future research, and clinical practice or policy development.	*Abstract.* The abstract summarizes the background and objectives, the literature considered, the main findings and challenges, and the implications and future directions of AI-enabled bioengineering.
INTRODUCTION	3	Rationale/background	Describe the rationale for the review in the context of what is already known.	*1. Introduction*. The rationale for an integrated narrative review is presented in the context of the rapid development and increasing integration of AI across bioengineering domains.
INTRODUCTION	4	Objectives	Specify the key question(s) identified for the review topic.	*1. Introduction*. The objectives are defined around the major Innovations, Challenges, and Future Directions of AI-enabled bioengineering,

				with particular attention to clinical and translational aspects.
METHODS	5	Research selection	Specify the process for identifying the literature search (e.g., years considered, language, publication status, study design, and databases of coverage).	*2.1. Design of the Review; Supplementary Material S1.1. Qualification Methodology; Supplementary Material S1.3. Literature Selection Process; Table A1. Search Strategies.* These sections describe the databases and thematic search strategies, the literature selection process, and the additional qualification methodology applied to the selected secondary literature.
DISCUSSION/SUMMARY	6	Narrative	Discuss: 1) research reviewed including fundamental or key findings, 2) limitations and/or quality of research reviewed, and 3) need for future research.	*6.1. Added Value and Role of the Review; 6.2. Interpretation of the Reviewed Evidence; 6.3. Limitations and Future Directions.* These sections interpret the reviewed evidence, consider its methodological and translational characteristics and limitations, and identify directions for future research.
DISCUSSION/SUMMARY	7	Summary	Provide an overall interpretation of the narrative review in the context of clinical practice and/or the Nutrition Care Process for registered dietitian nutritionists, clinical practice for other health professionals, policy development and implementation, or future research.	*6.3. Limitations and Future Directions; 7. Conclusions.* The overall interpretation emphasizes clinical and translational development, responsible implementation, future research, and the conditions required for reliable and sustainable use of AI-enabled bioengineering systems.

S1.3. Literature Selection Process

Three complementary literature searches were conducted using the thematic search keys corresponding to **Innovations, Challenges, and Future Directions**. The searches were performed in both PubMed and Scopus, **without imposing any temporal restrictions, and covered the literature available up to the date of manuscript submission**. After combining the retrieved records and removing duplicate records, **1,583 publications** remained for the subsequent selection process.The search strategy was specifically oriented toward the identification of **systematic reviews and meta-analyses**, which constituted the principal target and main evidence base of the secondary literature. Titles and abstracts were screened to identify publications potentially relevant to the scope and

objectives of the review. Following this initial screening, **347 publications** were retained for full-text assessment.

Although the search strategy was specifically focused on systematic reviews and meta-analyses, the retrieved literature also included other types of secondary publications. These included **scoping reviews, narrative reviews, umbrella reviews, and other particularly structured review articles**. Such publications emerged within the retrieved literature and were not automatically excluded when their content, structure, and methodological characteristics indicated that they could provide a relevant and informative contribution to the specific topic addressed.

The full texts were therefore examined to assess the relevance and potential contribution of the identified publications to the narrative synthesis. Particular attention was given to the thematic focus, the structure and methodological characteristics of the review, the breadth and relevance of the evidence considered, and its contribution to the specific area of the present review. Following full-text assessment, **126 publications** were retained for further consideration.

The publications retained at this stage were subsequently subjected to the qualification methodology described in Section S1.1. Following the overall selection and qualification process, **57 secondary publications** were included in the principal evidence base of the narrative synthesis.

https://doi.org/10.3390/bioengineering13101204
